# Nuclear magnetic resonance and surface-assisted laser desorption/ionization mass spectrometry-based serum metabolomics of kidney cancer

**DOI:** 10.1007/s00216-020-02807-1

**Published:** 2020-07-13

**Authors:** Joanna Nizioł, Krzysztof Ossoliński, Brian P. Tripet, Valérie Copié, Adrian Arendowski, Tomasz Ruman

**Affiliations:** 1 Faculty of Chemistry, Rzeszów University of Technology, 6 Powstańców Warszawy Ave, 35-959 Rzeszów, Poland; 2 Department of Urology, John Paul II Hospital, Grunwaldzka 4 St, 36-100 Kolbuszowa, Poland; 3 The Department of Chemistry and Biochemistry, Montana State University, Bozeman, MT 59717 USA

**Keywords:** Kidney, Cancer, Mass spectrometry, Biomarkers, Proton nuclear magnetic resonance

## Abstract

Kidney cancer is one of the most frequently diagnosed and the most lethal urinary cancer. Despite all the efforts made, no serum-specific biomarker is currently used in the clinical management of patients with this tumor. In this study, comprehensive high-resolution proton nuclear magnetic resonance spectroscopy (^1^H NMR) and silver-109 nanoparticle-enhanced steel target laser desorption/ionization mass spectrometry (^109^AgNPET LDI MS) approaches were conducted, in conjunction with multivariate data analysis, to discriminate the global serum metabolic profiles of kidney cancer (*n* = 50) and healthy volunteers (*n* = 49). Eight potential biomarkers have been identified using ^1^H NMR metabolomics and nine mass spectral features which differed significantly (*p* < 0.05) between kidney cancer patients and healthy volunteers, as observed by LDI MS. A partial least squares discriminant analysis (OPLS-DA) model generated from metabolic profiles obtained by both analytical approaches could robustly discriminate normal from cancerous samples (Q^2^ > 0.7), area under the receiver operative characteristic curve (ROC) AUC > 0.96. Compared with healthy human serum, kidney cancer serum had higher levels of glucose and lower levels of choline, glycerol, glycine, lactate, leucine, *myo*-inositol, and 1-methylhistidine. Analysis of differences between these metabolite levels in patients with different types and grades of kidney cancer was undertaken. Our results, derived from the combination of LDI MS and ^1^H NMR methods, suggest that serum biomarkers identified herein appeared to have great potential for use in clinical prognosis and/or diagnosis of kidney cancer.

**Electronic supplementary material:**

The online version of this article (10.1007/s00216-020-02807-1) contains supplementary material, which is available to authorized users.

**Figure:**
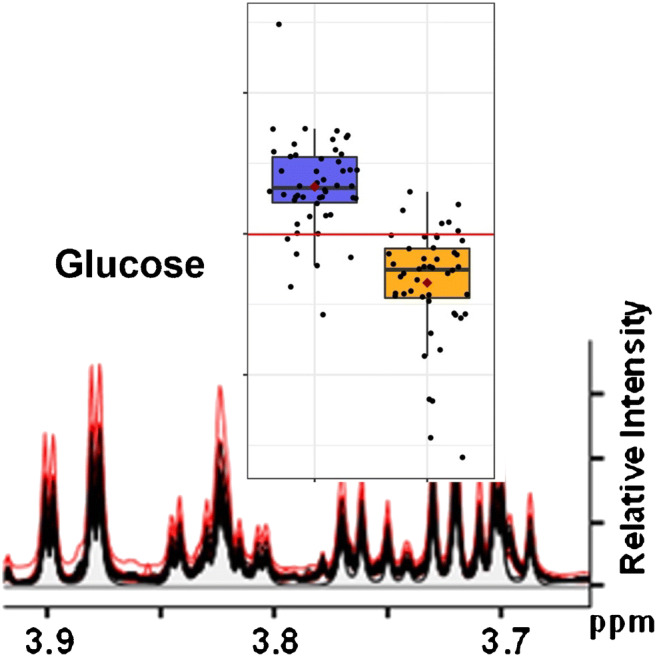
Graphical abstract

## Introduction

Kidney cancer is the third most frequently diagnosed cancer of the urinary tract in the world. In 2018, this disease affected over 400,000 individuals worldwide and is responsible for nearly 180,000 deaths annually [1]. An increased understanding of kidney cancer has shown that this is not a single disease, but rather originates from a number of different types in this organ, which is driven by differential gene expression, and characterized by different clinical trajectories and outcomes, histological manifestations, and responses to therapy [2]. Benign kidney tumors (non-cancerous) do not have the ability to spread (metastasize) to other parts of the body, while malignant (cancerous) tumors grow and spread out of control. There are various types of non-cancerous tumors of the kidney including adenoma, oncocytoma, and angiomyolipoma (AML). The most common malignant types of kidney cancer accounting for > 90% of cancers in this organ are renal cell carcinomas (RCC) including clear cell (ccRCC), chromophobe RCC (cRCC), and papillary RCC (pRCC), which originate from the renal tubular epithelial cells. The remaining subtypes of RCC, including duct carcinoma (CDC), angiomyolipoma (AML), or simple renal cyst (SRC), are very rare [3]. Nowadays, diagnosis of RCC is based on imaging procedures and more than 50% of RCC are diagnosed incidentally. In most cases, RCC is difficult to detect at an early stage due to the lack of characteristic symptoms such as triad of hematuria, low back pain, and flank mass symptoms. Most patients exhibit systemic symptoms including weight loss, anorexia, abdominal pain, and fever, and approximately one-third of patients with RCC have locally advanced or metastatic tumors beyond the kidney at the time of diagnosis [4]. The lack of adequate therapies at this stage, as well as the inherent resistance of this tumor to chemotherapy and radiotherapy, is associated with poor prognosis and high mortality rate. Less than 10% of patients with metastatic disease are alive 5 years after diagnosis [5]. Of the currently available localized RCC treatments, such as active surveillance or cryoablation and radiofrequency ablation, the most effective is still radical nephrectomy with nephron-sparing surgery at an early stage [2]. Despite this type of treatment, nearly one-third of patients experience disease recurrence after surgical resection.

The survival of advanced kidney cancer patients, for whom standard chemotherapy is not very effective, has improved with the advent of targeted therapy. The targeted drugs act against vascular endothelial growth factor (VEGF) and other important proteins (tyrosine kinases) that enable cancer cell growth and survival, but treatment response is varied; and at best, these targeted drugs can only slow the growth of the cancer for a time but do not actually cure kidney cancer. Specific targeting molecules, most of which are proteins, have been proposed (C-reactive protein (CRP), PTEN, carbonic anhydrase IX (CAIX), hypoxia-inducible factors (HIF-1α and HIF-1β), vascular endothelial growth factor (VEGF, CD44, E-cadherin, osteopontin, antigen Ki-67 and tumor protein p53), and monitoring their activity might generate a timely prognosis of the metastatic potential of RCC. However, these biomarkers suffer from low abundance and are not particularly specific to metastatic RCC, and using them as diagnosis of metastasized RCC is very limited [6]. In addition, numerous low molecular weight markers of kidney cancer have been proposed including lysophosphatidylcholines, phenylacetylglycine, ganglioside GM3, sphingomyelin, thromboxane, phenylacetylglycine, acetylphenylalanine, glycocholic acid, glycerophosphorylcholine, carnitine, lysophosphatidylethanolamines, tryptophan, tyrosine, ldl/vldl, lactate, choline, valine, leucine, isoleucine, glutamate, glutamine, lipids metabolites, carbohydrates metabolites, alanine, and creatine [7–11].

Unfortunately, though great efforts have been undertaken in the past decades, there are still no acknowledged robust biomarkers available for diagnosis or prognosis of RCC cancers. In order to create more effective therapies, additional kidney cancer biomarker research is needed, and has important clinical significance especially for early detection, diagnosis, as well as guiding treatment interventions, monitoring treatment effectiveness, identifying relapse, and elucidating the molecular processes underlying RCC disease states.

Over the past decade, sensitive analytical methods have been developed, enabling to better understand the metabolic changes underlying kidney cancer phenotypic changes. These analytical platforms include ^1^H nuclear magnetic resonance (NMR) [12], liquid chromatography-coupled mass spectrometry (LC-MS) [8], gas chromatography-coupled mass spectrometry (GC-MS) [13], matrix-assisted laser desorption/ionization surface (MALDI) [14], desorption electrospray ionization mass spectrometry (DESI MS) [15], and surface-enhanced laser desorption/ionization mass spectrometry (SELDI MS), which when employed together enable to achieve the most comprehensive screening of cancer metabolomes [16].

NMR and MS combined with multivariate statistical data analysis are the two most powerful and commonly used analytical methods for the rapid, noninvasive, and simultaneous measurements of endogenous metabolites present in biological fluids and tissues [17]. These two techniques are highly complementary; NMR is quantitative, reproducible, and does not require extensive steps for sample preparation, such as separation or derivatization. MS is, on the other hand, extremely sensitive and allows for the detection of thousands of mass spectral features corresponding to numerous metabolites, and can be undertaken using very small amounts (a few mg) of sample. Thus, utilization of both MS and NMR methods in concert expands metabolite coverage and enables the comprehensive analysis of a wide range of metabolome profiles [18].

Metabolic profiling of biofluids, especially serum, generates a biochemical fingerprint of small molecule metabolites, and allows for identification and characterization of potential biomarkers associated with cancer. Since genetic studies has shown that RCC is a metabolic disease, a growing number of studies is focusing on profiling of tissue, serum, plasma, and urine samples of kidney cancer patients. However, relatively low number of NMR metabolomics studies have been undertaken to characterize the serum metabolomes of kidney cancer patients in recent years [19].

Lin and co-workers are pioneers in the analysis of the serum of patients with kidney cancer using mass spectrometry. In 2010, they employed separately two MS-based techniques to analyze serum samples from 31 kidney cancer patients and 20 healthy volunteers. The authors found a total of 71 variables useable as potential markers but the identification of chemical compounds was only successful for only a few of them [7]. A year later, Lin et al. utilized LC-MS-based metabolomics profiling to evaluate differential levels of compounds present in serum of 33 RCC patients and 25 healthy volunteers. Application of two different chromatographic columns and combination of data sets, they found 30 potential serum biomarkers [20]. In 2008, Gao et al. performed metabolic profiling of serum from 74 patients with kidney cancer and 55 controls using proton nuclear magnetic resonance (^1^H NMR) spectroscopy and found 18 metabolites significantly differentiating between three groups: (i) RCC patients and controls; (ii) RCC patients with metastases and without metastases; and (iii) RCC patients before and after nephrectomy [21]. Zira et al. applied ^1^H NMR spectroscopy to analyze serum metabolome of plasma in 32 RCC patients and 13 controls and found 17 metabolites that can distinguish RCC patients form controls [9]. In another study, Jobard et al. conducted a metabolomics analysis of serum samples from 171 patients with mRCC participating in clinical trial to identify metabolic signatures associated with targeted therapies using ^1^H NMR [10]. ^1^H NMR-derived metabolomics profile of serum samples from 104 participants was studied by Zheng et al. in order to prepare RCC predictions. They revealed that cluster of 7 metabolites (alanine, creatine, choline, isoleucine, lactate, leucine, and valine) can be used for the early diagnosis of RCC [11]. Moreover, studies focusing on the metabolite profiling of tissues [12, 22, 23] and urine [24, 25] from patients with kidney cancer have been reported. Recently, comprehensive review regarding metabolic profiling of samples from RCC patients has been published [19].

In the current work, the metabolomics analysis of 50 serum samples from patients with kidney cancer and 49 healthy subjects serving as controls was undertaken using two orthogonal analytical methods: high-resolution ^1^H NMR and laser desorption/ionization MS using a 109-silver nanoparticle-enhanced steel target platform (^109^AgNPET LDI MS) [26]. The value of the ^109^AgNPET LDI MS approach for metabolomics has been demonstrated in the analysis of metabolites in plant, animal, and human tissues [27]. Both multivariate statistical data and quantitative analysis of metabolite profiles were employed to assess whether patient cohorts could be separated from control individuals, based on distinct metabolite patterns observed in serum samples, and using 1D ^1^H NMR untargeted metabolomics.

## Materials and methods

### Materials and equipment

Silver-109 (min. 99.75% of ^109^Ag) isotope was purchased from BuyIsotope (Sweden) and transformed to trifluoroacetate salt using commonly known methods (involving dissolving in HNO_3_, precipitation of ^109^AgOH, and reaction with trifluoroacetic acid) and recrystallized from tetrahydrofuran/hexane system. 2,5-Dihydroxybenzoic acid (DHB) was purchased from Aldrich. Steel targets were locally machined from H17 stainless steel. All solvents were of HPLC quality, except for water and methanol (LC-MS grade, Fluka). The silver-109 nanoparticles were synthesized on the surface of steel targets as described in our publication [27].

### Collection of serum samples

The study protocol was approved by local Bioethics Committee at the University of Rzeszow (Poland) (permission no. 2018/04/10). We confirm that all research was performed in accordance with relevant guidelines and regulations. Specimens and clinical data from patients involved in the study were collected with informed consent. Blood samples were obtained from fifty patients with kidney cancer and 49 age- and sex-matched healthy control subjects, following detailed clinical questioning at John Paul II Hospital in Kolbuszowa (Poland). All laboratory test results (complete blood count, kidney function tests, CRP, urine analysis, bleeding profile) were within normal ranges. Serum samples from 50 patients (20 female, 30 male, age range 36–87, average age 69) with kidney cancer and 49 healthy control subjects were collected. The majority of patients (*n* = 33, one patient had two tumors) had stage T1 disease, four patients had stage T2, ten patients had stage T3, and one patient had stage T4. In three patients, the stage of the disease could not be determined. Among tumors diagnosed, there were 33 clear cell renal cell carcinomas (ccRCC), 4 oncocytomas, 4 angiomyolipomas (AML), 2 chromophobe renal cell carcinomas (chRCC), 2 papillary renal cell carcinomas (pRCC), 1 collecting duct carcinoma (CDC), 1 simple renal cyst (SRC), and 1 tubulocystic renal cell carcinoma (TCRC) according to the 2016 *WHO Classification of Tumors of the Urinary System and Male Genital Organs*. Most of the diagnosed cancers were malignant (*n* = 41), but few patients (*n* = 7) had benign (non-cancerous) kidney tumors. In this study, benign tumors of the kidney include oncocytoma and angiomyolipoma, while other types of tumors are considered malignant. In addition, one patient had a lung adenocarcinoma metastasis. The majority of patients (*n* = 33, with one patient having two tumors) had stage T1 disease, four patients had stage T2, ten patients had stage T3, and one patient have stage T4. In three patients, the stage of the disease could not be determined. The pathological and clinical characteristics of the patients are presented in the Electronic Supplementary Material (ESM, Table S1). About 2.6 mL of blood was drawn from each participant. After approximately 1 h at room temperature, the samples were centrifuged at 3000 rpm for 10 min. The serum was separated and stored at − 60 °C until further use.

### Preparation of serum samples

Prior to NMR analysis, serum samples were thawed at 4 °C, then centrifuged at 12000×*g* for 5 min at 4 °C to remove cells and other precipitated material. A volume of 900 μL of acetone was added to 300 μL of resulting supernatants. After vortexing for 1 min, the solutions were incubated at room temperature for 20 min followed by 30 min at − 20 °C, and then centrifuged at 6000×*g* for 5 min at 4 °C. Next, 800 μL volumes of clarified supernatants were transferred to a new polypropylene tube. The pellets were re-suspended in 500 μL of acetone-H_2_O mixture (3:1, v/v) and vortex vigorously. The samples were subjected to centrifugation at 12000×*g* for 10 min at 4 °C. The supernatants from the pellet wash were combined with the supernatants from the first spin. Finally, from 990 μL of resulting samples, 50 μL was taken and used for ^109^AgNPET LDI MS analysis. The rest of the sample was dried to complete dryness in a SpeedVac vacuum concentrator, with no heat. Dried extracts were re-suspended in 600 μL of NMR buffer consisting of 25 mM NaH_2_PO_4_/Na_2_HPO_4_, 0.4 mM imidazole, 0.25 mM 4,4-dimethyl-4-silapentane-1-sulfonic acid (DSS) in 90% H_2_O/10% D_2_O, pH 7.0. Following re-suspension, samples were centrifuged at 21,000 rpm for 1 min to pellet insoluble debris, and then transferred to 5 mm NMR tubes for NMR metabolomics analysis.

### NMR spectra acquisition and preprocessing

1D ^1^H NMR spectra were collected at 298 K (25 °C) using a Bruker 600 MHz (^1^H Larmor frequency) AVANCE III solution NMR spectrometer, equipped with a SampleJet automatic sample loading system, a 5 mm triple resonance (^1^H, ^15^N, ^13^C), liquid-helium-cooled TCI NMR cryoprobe, and Topspin software (Bruker version 3.6). 1D ^1^H NMR spectra acquisition was performed using the Bruker-supplied excitation sculpting (ES)-based “zgesgp” pulse sequence, and NMR spectra were recorded with 256 scans and a ^1^H spectral window of 7211.538 Hz. Free induction decays (FIDs) were collected with 64 K data points and a dwell time interval of 69.33 μsec, amounting to a data acquisition time of 4.54 s. Relaxation delay (D1) times between acquisitions were set to 2 s, resulting in an overall 6.5-s delay between scans. DSS chemical shift referencing and phase correction of 1D ^1^H NMR spectra were conducted using Topspin software (Bruker version 3.6).

For verification of Chenomx-annotated metabolites, 2D ^1^H-^1^H total correlation spectroscopy (TOCSY) spectra were acquired for representative samples using the Bruker-supplied “mlevphpr.2/mlevgpph19” pulse sequences (256 × 2048 data points, 2-s relaxation delay, 32 transients per FID, 1H spectral window of 6602.11 Hz, 80 ms TOCSY spin lock mixing period). 2D ^1^H-^1^H TOCSY spectra were processed using Topspin software (Bruker version 3.6).

### NMR data analysis

Further processing of 1D ^1^H NMR spectra and metabolite profiling analyses were conducted using the Chenomx NMR Suite software (version 8.1; Chenomx Inc., Edmonton, Alberta, Canada). Baseline correction of NMR spectra following import of preprocessed “1r” NMR spectral files into Chenomx software was performed using the automatic cubic spline function in Chenomx, and subsequent manual breakpoint adjustment to obtain a flat, well-defined baseline, following recommendations from Chenomx application notes and previously reported methods [28]. ^1^H chemical shifts were referenced to the 0.0 ppm DSS signal, and the ^1^H NMR signals arising from imidazole were used to correct for small chemical shift changes due to slight variations in sample pH. Metabolite identification and quantification were performed by fitting the 1D ^1^H spectral splitting patterns, chemical shifts, and spectral intensities to reference spectral patterns of small molecules using the Chenomx small molecule spectral database for 600 MHz (^1^H Larmor frequency) magnetic field strength NMR, and manually peak-based fits, where adjustments were made to achieve optimal spectral pattern fits for compound peak cluster location and intensity. An internal (0.25 mM DSS) standard was used for metabolite quantitation. Although pulse sequences utilizing the “ZGESGP” pulse sequence scheme suppress proton signals around the water region to a greater extent than the NOESYPR pulse sequence (i.e., 1D NOESY with presaturation during relaxation and mixing time), the relative intensities observed for these particular ^1^H signals whose resonance frequencies are close to that of the water ^1^H are largely identical to those seen using the noesypr1d sequence. To adjust for minor differences between 1D ^1^H spectra acquired using “zgesgp” versus “noesypr1d” pulse sequences, we have created our own in-house “zgesgp”-acquired 600 MHz metabolite library using pure standards and the “Compound Builder” module of Chenomx NMR Suite program (version 8.1), as described previously in the operational manual.

### MS sample preparation

Volume of 0.3 μL of each sample was placed on a ^109^AgNPET and allowed to dry at room temperature, then target was inserted into a MALDI ToF/ToF mass spectrometer.

### MS spectra acquisition and preprocessing

Laser desorption ionization mass spectrometry imaging (LDI-MSI) experiments were performed using a Bruker Autoflex Speed time-of-flight mass spectrometer in positive-ion reflectron mode. The apparatus was equipped with a SmartBeam II 1000 Hz 355 nm laser, with a laser impulse energy of approximately 100–190 μJ, laser repetition rate of 1000 Hz, and deflection set on *m/z* lower than 80, with a *m/z* range of 80–2000 Da. Spectrum for each extract contained data from 20k laser shots with a default random walk applied (random points with 50 laser shots). All spectra were calibrated with the use of silver ions (^109^Ag^+^ to ^109^Ag_10_^+^). The first accelerating voltage was held at 19 kV, and the second ion source voltage was held at 16.7 kV. Reflector voltages used were 21 kV (the first) and 9.55 kV (the second). FlexAnalysis 4.0 software was used for data processing and analysis.

### Multivariate statistical analysis

A total of 99 ^1^H NMR spectra were recorded, corresponding to serum samples of 50 patients with kidney cancer and 49 healthy controls, and 100 LDI MS spectra were recorded for the same 50 patients with kidney cancer and 49 healthy controls, and resulting data were subjected to multivariate data analysis. Both NMR and MS data sets were analyzed using multivariate data analysis to reveal metabolic changes in the serum of patients with kidney cancer with respect to healthy controls.

Metabolite concentrations normalized by sum were further log-transformed to ensure a Gaussian distribution of the data and auto-scaled (i.e., mean centered and divided by the standard deviation), prior to statistical analysis, including principal component analysis (PCA), partial least squares discriminant analysis (PLS-DA), and orthogonal partial least squares discriminant analysis (OPLS-DA), which was accomplished using the MetaboAnalyst software 4.0 [29]. The overall quality of the PLS-DA models was assessed by examining cross-validation parameters specifying accuracy, predictive ability of the model (*Q*^2^), and goodness of fit (*R*^2^). Variable Importance in the Projection (VIP) plots and S-plots were generated to identify metabolites whose level changes were most significantly responsible for group separation. Metabolites with VIP scores > 1, |p| > 0.05 (magnitude), and |p(corr)| > 0.5 (reliability) were considered to be potential biomarkers that distinguish kidney cancer patients from healthy controls. In this work, 10-fold cross validations were used to define the number of latent variables (PLS components) in the model. To test the accuracy of multivariate models and minimize the possibility that the observed separation in the PLS-DA and OPLS-DA is due to chance (*p* < 0.05), permutation tests were performed with 2000-fold repetition. Statistical significance of metabolite level differences was assessed using unpaired parametric *t* test with Mann-Whitney and Bonferroni correction. *p* values and false discovery rates (FDR; *q* value) less than 0.05 were considered statistically significant. Moreover, receiver operating curve (ROC) analysis was done to evaluate the diagnostic value of selected metabolites. Standard chemometrics tools such as 2D PCA and PLS-DA analysis were also used to assess metabolic profile similarities and differences between cancer types (malignant and benign) and grades (grades 1, 2, and 3). An approximation was used whereby all malignant renal tumors like ccRR, chRCC, pRCC, CDC, SRC, and benign kidney tumors (oncocytoma and AML) were grouped together, expecting that changes in serum metabolite levels in tumor types would follow similar trends.

## Results

In this study, we characterized the metabolic profiles of patients suffering from kidney cancer, in an effort to develop serum-specific metabolomics signatures for early and specific detection of kidney cancer. For this purpose, we recorded high-resolution 1D ^1^H NMR spectra on 99 total (50 RCC and 49 controls) metabolite extracts from serum samples.

**Fig. 1:**
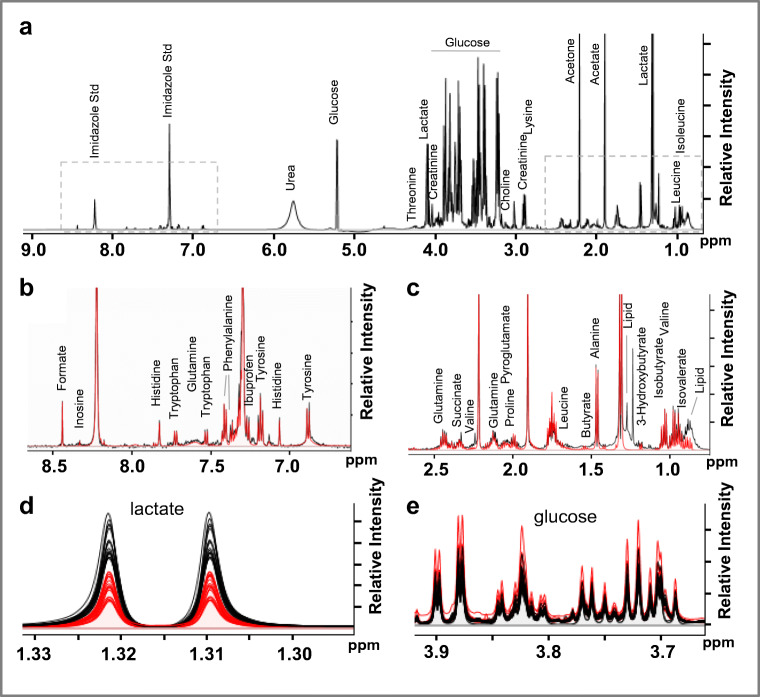
**a** Characteristic 1D ^1^H NMR spectrum of a protein-free metabolite extract mixture obtained from a serum sample of a kidney cancer patient, recorded on MSU 600 MHz (14 Tesla) solution NMR spectrometer. NMR signals of specific metabolites are labeled. **b**–**c** Expanded regions of the spectrum shown in **a** with NMR resonances corresponding to specific metabolites indicated. The chemical shift ranges of the expanded spectral regions shown in panels **b** and **c** are indicated by dashed line boxes in the full spectrum shown in **a**. The expanded spectra shown in **b** and **c** depict the spectral overlay of the “fitted” ^1^H NMR spectrum (red trace), which was generated using the Chenomx data processing and analysis software, with the original NMR spectra depicted by the black traces. **d**, **e** Expanded NMR spectral regions corresponding to ^1^H chemical shift ranges of 1.33–1.30 ppm (**d**) and 3.93–3.68 ppm (**e**), with a spectral overlay of 15 serum metabolic profiles obtained from healthy patients depicted in black (black spectral traces) and kidney patients in red (red spectral traces). These spectral regions illustrate the typical NMR signals observed for lactate and glucose, respectively. The intensity-normalized spectral overlays shown in **d** and **e** clearly indicate that lactate levels are lower and glucose levels are higher in the serum profiles of kidney patients (red) compared with healthy controls (black)

Analysis of ^1^H NMR spectral patterns resulted in the resonance assignment and quantitation of 43 metabolites in each serum sample. Representative ^1^H NMR spectra of serum samples from healthy subjects and kidney cancer patients are shown in Fig. 1, along with metabolites identified.

Significant differences were observed in levels of individual metabolites when the metabolic profiles of serum samples from patients with kidney cancer were compared with those of healthy controls. Multivariate and univariate statistical analyses of these metabolite patterns were performed to assess similarities and differences in serum metabolomes from the two different groups. This analysis also enabled us to identify a panel of polar small molecules whose levels are altered significantly in kidney cancer.

### Distinguishing between kidney cancer and control samples by ^1^H NMR

**Fig. 2:**
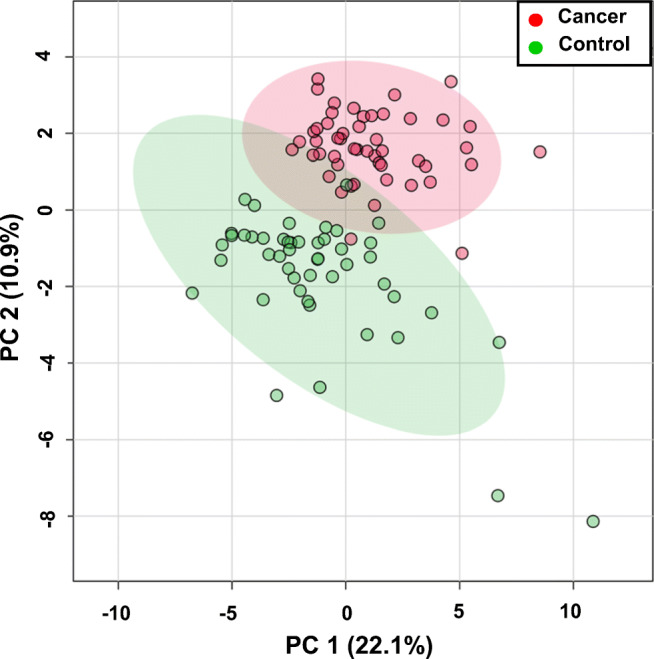
Unsupervised two-dimensional principal component analysis (2D-PCA) score plot generated from the analysis of the metabolic characteristic of serum samples from kidney cancer patients (red) and healthy control individuals (green), with principal components 1 (PC1) and 2 (PC2) accounting for 22.1% and 10.9% of the variance, respectively

Multivariate analysis was conducted based on metabolite concentration data obtained by NMR. Unsupervised principal component analysis (PCA) was performed to search for trends in group separation and to identify potential outliers. The results are presented as 2D PCA scores plots, where each colored dot represents a serum sample from a cancer patient (red) or healthy control (green) in Fig. 2.

**Fig. 3:**
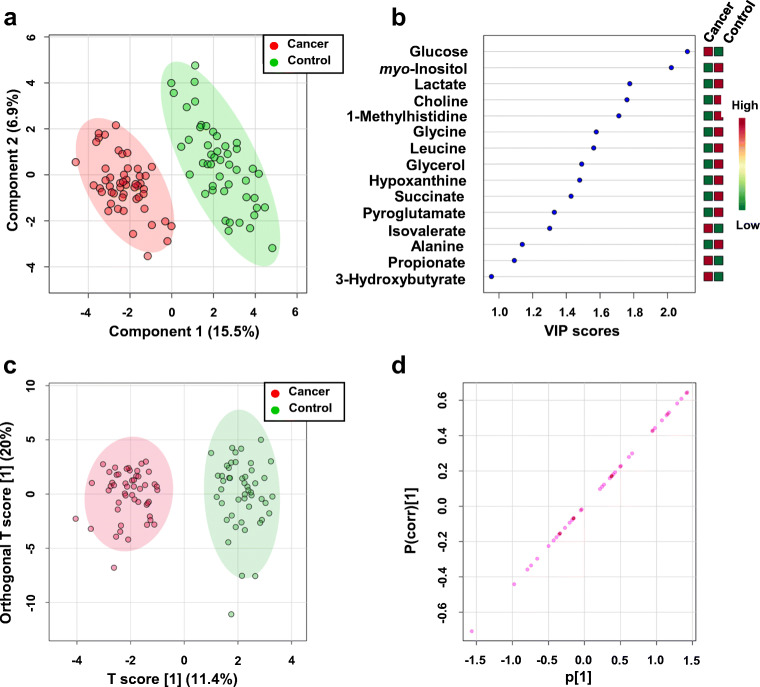
Supervised 2D PLS-DA and OPLS-DA analysis of serum metabolite profiles identified by ^1^H NMR. **a** 2D PLS-DA score plot revealing that the kidney cancer (red circles) patient group separates clearly from healthy controls (green circles), with PC1 and PC2 accounting for 15.5% and 6.9% of the variance, respectively, and large green and red ellipses representing the 95% confidence intervals of the group clustering. And **b** associated Variable Importance in Projection (VIP) scores ranking the most important metabolites (VIP > 1) that contribute to the separation in the PC1 dimension of the two sample groups. **c** OPLS-DA score plot and **d** loading S-plot

The 2D PCA score plot shown in Fig. 2 reveals a good separation between the cancer patient and healthy control groups, with PC1 and PC2 accounting for 22.1% and 10.9%, respectively, of the variance. Next, we performed supervised partial least squares discriminant analysis (PLS-DA) and orthogonal partial least squares discriminant analysis (OPLS-DA) to assess the extent of differences between the two groups that may reveal potential biomarker indicators of the two different physiological states. The classification from 2D-PLS-DA score plots and VIP score results is shown in Fig. 3.

Parameters employed to evaluate the robustness and validity of the PLS-DA modeling include assessment of *R*^2^ and *Q*^2^ values and data permutation tests. This analysis resulted in an *R*^2^ value of 0.92, a *Q*^2^ of 0.86, an accuracy of 0.99, and *p* values < 5E−4 for the data shown in Fig. 3, demonstrating that the group separation observed is real, and that the PLS-DA model is robust and valid (Fig. S1, see ESM). Subsequently, fourteen potential metabolite biomarkers were pre-selected based on VIP scores ranking as shown in Fig. 3b. Additional OPLS-DA was conducted to improve the model’s effectiveness and to extract variable information that facilitates sample classification. The OPLS-DA score plot presented a clear discrimination between two groups as shown in Fig. 3c. To evaluate statistical parameters of this model, 2000-permutation tests were conducted. The resulting values of *R*^2^Y and *Q*^2^ were respectively 0.92 (*p* value < 5E−04 (0/2000)) and 0.865 (*p* value < 5E−04) giving an indication of good fitness and predictability of the two-class model (Fig. S1, see ESM). The metabolites that are significantly varying between the two-class models were identified using the loadings of S-plot of OPLS-DA data based on the criterion that |p(corr)| > 0.5 (Fig. 3d). Seven variables were positively correlated to group separation showing +p(corr) [1] > 0.5 and one negatively correlated showing −p(corr) [1] < − 0.5.

Biomarker candidates were further subjected to univariate *t* test analysis to check the significance of altered levels of these metabolites in serum samples of cancer patients versus healthy controls. In total, 8 of the identified metabolites were considered to be present at statistically significantly differential concentrations (*p* < 0.05; *q* < 0.05, VIP > 1 and |p(corr)| > 0.5). Mean metabolite concentrations with all relevant statistical parameters are summarized in Table S2 (see ESM). To further assess the predictive value of these metabolites to discriminate between the serum profiles of kidney cancer patients and controls, we performed multivariable ROC curve analysis, using the differential concentrations of this subset of metabolites. The quality of the ranking represents the area under the curve (AUC). The closer AUC approaches a value of 1, the better the discrimination performance of the ROC curve. The classifier is of no practical utility when AUC reaches 0.5, as this indicates that subject classification is random. In our study, all previously selected metabolites have an area under the curve (AUC) > 0.8 were found to include choline, glucose, glycerol, glycine, lactate, leucine *myo*-inositol, and *pi*-methylhistidine (Table 1).

**Table 1:** Summary of relative concentration changes of potential metabolite markers, as revealed from ^1^H NMR spectral analyses of serum samples from kidney cancer patients and healthy control volunteers

No.	Metabolite	AUC	VIP	p(corr)	*p* value^a^	Fold change^b^				
						Cancer vs. control	Malignant vs. benign	G1 vs. G2	G1 vs. G3	G2 vs. G3
1	Choline	0.84	1.76	0.609	1.04E−08	0.7	1.0	0.9	0.9	1.1
2	Glucose	0.93	2.12	− 0.708	1.85E−13	1.7	1.1	0.8	0.8	1.0
3	Glycerol	0.81	1.49	0.516	1.48E−07	0.7	1.0	0.8	0.7	0.9
4	Glycine	0.80	1.58	0.531	1.93E−07	0.8	0.9	1.0	1.0	1.0
5	Lactate	0.83	1.78	0.641	2.10E−08	0.7	0.7	1.0	1.2	1.2
6	Leucine	0.82	1.56	0.522	8.23E−08	0.8	1.0	1.0	0.9	1.0
7	*myo*-Inositol	0.98	2.02	0.645	3.11E−16	0.4	1.1	1.1	1.0	1.0
8	1-Methylhistidine	0.80	1.71	0.582	2.72E−07	0.3	1.3	0.5	1.0	2.0

^a^*p* value determined from Student’s *t* test with Welch’s correction; ^b^Calculated from the concentration mean values of each group; *G* grade of kidney cancer

**Fig. 4:**
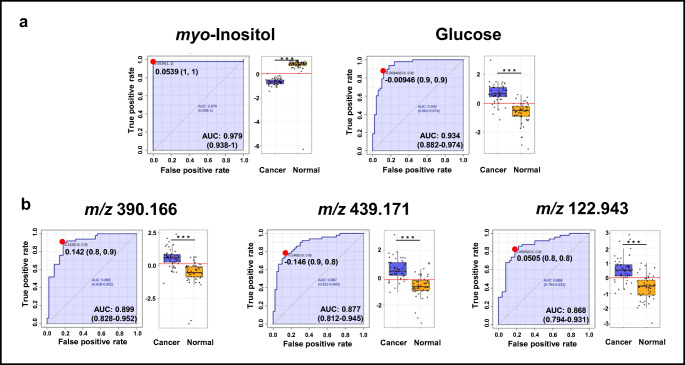
ROC curve analysis for potential biomarkers predicted by classical univariate analysis derived from **a**^1^H NMR data and **b**^109^AgNPET LDI MS data. The left-hand side of each panel indicates ROC curve for a particular metabolite, with 95% confidence interval (shadowed) and the solid red dot indicating the optimal cutoff, associated with sensitivity and specificity values. The right-hand side of each panel depicts the distribution of metabolite level values observed in control and kidney cancer serum samples. The horizontal red line in the graphs indicates the cutoff point

Among these metabolites, best results with highest significant were achieved for the metabolite *myo*-inositol (AUC = 0.979, specificity = 1, and sensibility = 1), followed by glucose (AUC = 0.934, specificity = 0.9, and sensibility = 0.9), choline (AUC = 0.837, specificity = 0.9, and sensibility = 0.7), and lactate (AUC = 0.833, specificity = 0.8, and sensibility = 0.8). The distribution of concentration values of individual metabolites in control and cancer serum samples is shown in Fig. 4a (the best results) and in Fig. S2 (see ESM).

Ten metabolites exhibiting the highest AUC > 0.8 in the ROC curve generated (Fig. S3, ESM) were used to assess the quality of this diagnostic model. The classification model for selected metabolites was built using analysis tools available in the MetaboAnalyst software based on the random forest algorithm. An excellent classification was obtained with AUC of 0.963, with a confidence interval (CI) from 0.922 to 1. This result suggests that eleven specific metabolites could be used as diagnostic biomarkers that separate serum samples from kidney cancer patients and control groups with high specificity and sensitivity.

### Distinguishing between grade and type of kidney cancer in ^1^H NMR dataset

**Fig. 5:**
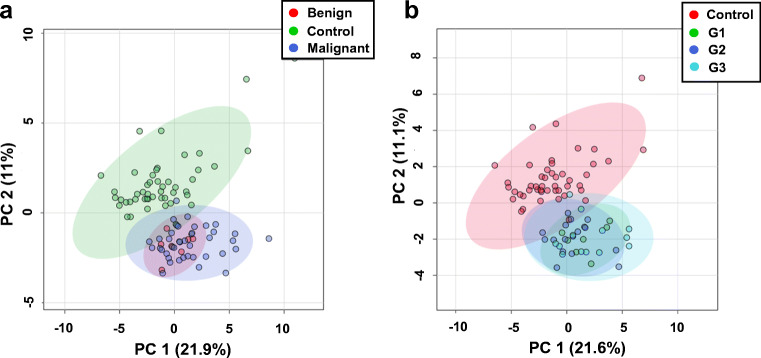
Unsupervised 2D principal component analysis (PCA) score plots of NMR data. **a** Patients with malignant (green circle) and benign (red circle) kidney cancer and healthy volunteers (blue circle). **b** Patients with grade 1 (green circle), grade 2 (blue circle), and grade 3 (turquoise circle) kidney cancer and healthy volunteers (red circle)

To demonstrate whether metabolite fingerprinting of serum extracts using ^1^H NMR metabolomics can significantly differentiate between kidney cancer tumor types (malignant and benign) and healthy control subjects, we assessed whether the 8 most significant metabolites identified in our PCA and PLS-DA analyses can also serve to distinguish between the different grades of kidney cancer tumors (grades 1–3) and the healthy control groups. A 2D-PCA score plot highlighting the extent of the separation three groups (malignant, benign kidney cancer, and healthy volunteers) based of distinct metabolic serum is shown in Fig. 5a, while Fig. 5b displays the 2D-PCA score plot of four groups that were classified according to kidney cancer grades, with grade 1, grade 2, grade 3 shown in green, dark blue, and cyan, respectively, and healthy volunteers in red.

**Fig. 6:**
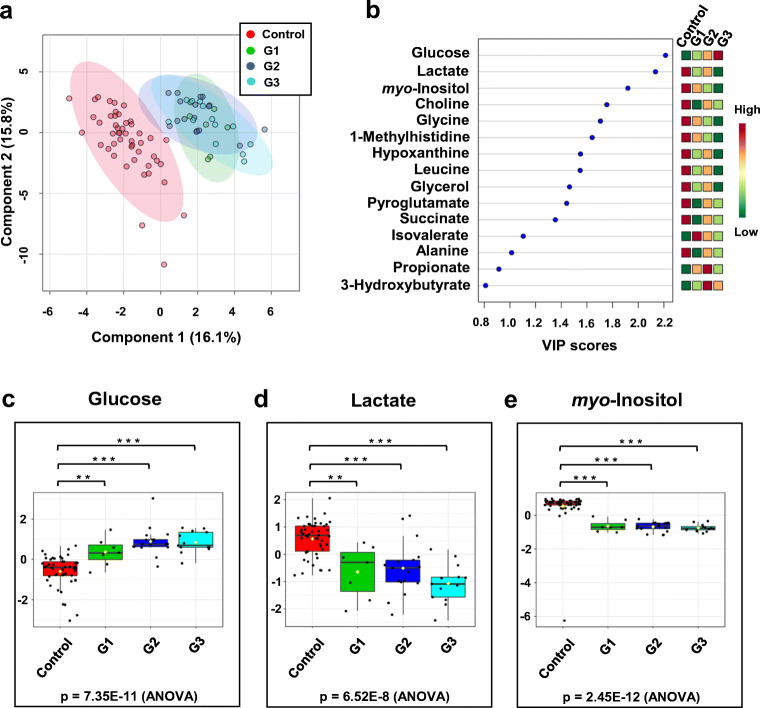
PLS-DA analysis based on metabolite profiles obtained from 1D ^1^H NMR spectra of patients with serum samples from kidney cancer patients varying in cancer tumor severity and healthy volunteers. **a** 2D-PLS-DA score plot assessing the extent of separation between different grades of kidney cancer (grade 1—green circle; grade 2—blue circle; grade 3—turquoise circle) and healthy volunteers (red circle). **b** Metabolite ranking according to the VIP score plot, resulting from the separation in PC1 observed in the 2D-PLS-DA plot. **c**–**e** Box-and-whisker plots for selected three panels of metabolites whose normalized concentration changes between the different tumor grades

**Fig. 7:**
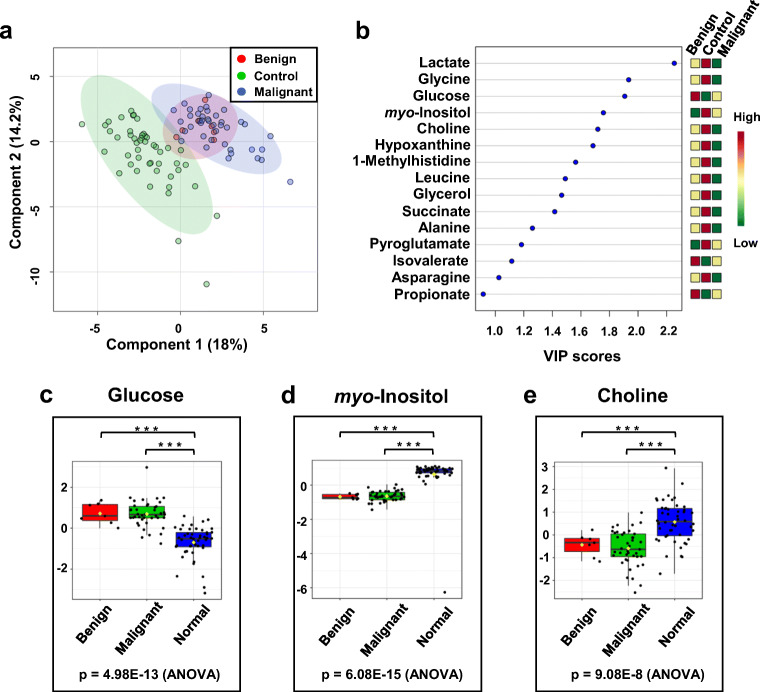
PLS-DA analysis based on metabolite profiles obtained from 1D ^1^H NMR spectra of patients with serum samples from kidney cancer patients varying in cancer tumor severity and healthy volunteers. **a** 2D-PLS-DA score plot highlighting the separation between malignant (green) and benign (red) kidney cancer and healthy volunteer (blue) groups. **b** Metabolite ranking according to VIP scores, with VIP > 1 indicating that the select metabolites contribute most to the separation between the groups. The colored boxes on the right-hand side of the VIP score plot denote relative metabolite concentrations between the different groups. **c**–**e** Box-and-whisker plots for selected three panels of metabolites whose normalized concentration changes between the different tumor type

Both 2D PCA score plots shown in Figs. 5a and b do not reveal significant group separation between the groups classified by different tumor grades, at least based on serum metabolome profiles as characterized by ^1^H NMR. This suggests that metabolic patterns for those groups are not easily separable by just simply assessing differences in polar metabolite levels in serum samples. Similarly, 2D-PLS-DA analysis could not distinguish clearly between tumor types and grades, as illustrated in Fig. 6a and 7 a; however, these groups were clearly distinct from the normal (control group), as indicated by the VIP score plots shown in Figs. 6b and 7b and the metabolites that are most responsible for the PLS-DA score plot patterns observed.

Validation parameters indicated that the group separation observed in the 2D-PLS-DA models were shown significant (*p* < 0.05) (ESM Fig. S4). Comparing concentration differences of selected metabolites extracted from serum samples of patients with various grades of kidney cancer suggested trends in metabolite level changes that appeared to track with kidney cancer grades, as shown in Table 1 and Figs. 6c–e and Fig. S5 (see ESM).

Data shown in Fig. 6c and d and Table 1 indicated that glucose and lactate may be considered indicators of the tumor grade.

Analysis of the selected metabolite concentration changes in a given type of cancer, i.e., benign versus malignant reveals higher levels of lactate in the serum samples of kidney cancer patients with a benign tumor compared with those whose tumor was malignant one. The trends are the opposite for 1-methylhistidine whose concentration is more elevated in the malignant cancer group compared with benign (Table 1, Fig. 7c–e and Fig. S6 in ESM).

### Metabolic profile of serum in kidney cancer with ^109^AgNPET LDI MS

**Fig. 8:**
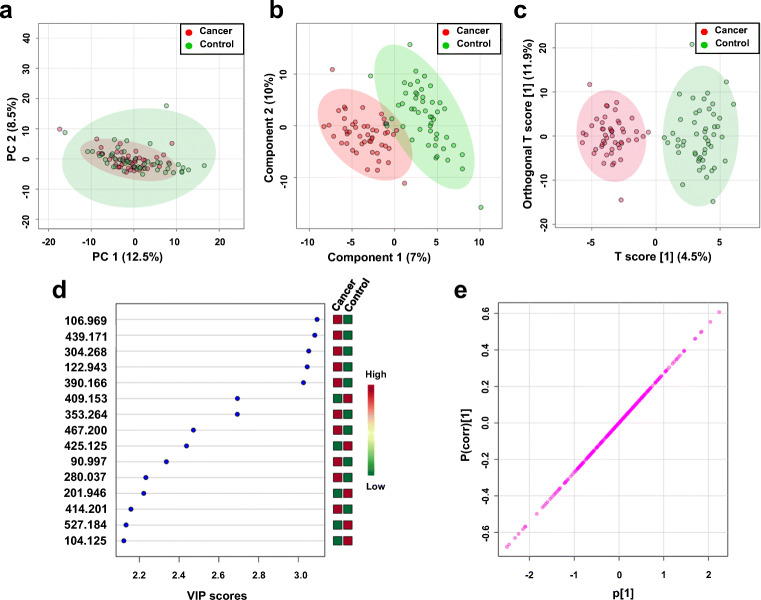
**a** Principal component analysis (2D-PCA) score plot between kidney cancer and control group based on ^109^AgNPET LDI MS metabolic profiles. **b** 2D-PLS-DA score plot and **c** OPLS-DA score plot based on ^109^AgLDI MS spectra of serum extracts obtained from patients with kidney cancer or healthy volunteers. **d** Key first 15 MS features according to the VIP-parameter (> 1). The colored boxes on the right indicate the relative concentrations of these metabolites in each group. **e** OPLS-DA loading S-plots

The peak intensity data from LDI MS spectra for metabolite extracts of serum samples was subjected to multivariate data analysis. 2D-PCA, 2D-PLS-DA, and OPLS-DA score plots were generated for the entire data set. As shown in Fig. 8a, the 2D-PCA score plot of LDI MS mass spectral features highlights a separation trend between the two groups, indicating that, although subtle, there exist inherent metabolic profile differences between kidney cancer and healthy controls.

Results from PLS-DA analysis as shown in Fig. 8b also clearly demonstrated a good separation between those two groups, which was actually more pronounced than the one identified from the NMR metabolite profiles. The goodness of fit and cross-validated predictive ability for the PLS-DA models for healthy control versus cancer group was reflected in the values of *R*^2^ = 0.90, *Q*^2^ = 0.70, accuracy = 0.89, with a permutation test also indicating PLS-DA model robustness and high predictability value of the model (*p* < 0.012, Fig. S7, see ESM). Based on the statistical analysis of the MS data, mass spectral features (*m/z* values) that contributed most to group separation were considered significant with *p* and *q* values < 0.05. As a result, 62 *m/z* variables were detected as potential serum discriminators of kidney cancer versus controls, using ^109^AgNPET LDI MS (Table S3, see ESM). To assess the potential of these *m/z* features to represent robust biomarkers of cancer phenotypes, VIP score plots were employed to assess their degree of importance (Fig. 8d). Only variables with VIP above 1.0 were considered important. The variables whose intensity changes were not significantly different between the patients and the controls (*p* > 0.05; *q* > 0.05, VIP < 1) were removed from this analysis. Next, OPLS-DA was applied to the MS dataset to reveal the features that had the greatest contribution to the discrimination between serum samples from kidney cancer and controls (Fig. 8c). The OPLS-DA score plot presented a clear discrimination between two groups. To evaluate the statistical robustness of this model, 2000-permutation tests were conducted. The resulting values of *R*^2^Y and *Q*^2^ were respectively 0.902 (*p* value < 5E−04 (0/2000)) and 0.708 (*p* value < 5E−04), giving an indication of good fitness and predictability of the two-class model (Fig. S7, see ESM). Potential features for group separation were subsequently identified by analyzing the S-plot of OPLS-DA data based on the criterion that |p(corr)| > 0.5 and |p| > 0.05 (Fig. 8e). The loading S-plot revealed that three variables were positively correlated to group separation showing +p(corr) [1] > 0.5 and six negatively correlated showing −p(corr) [1] < − 0.5.

Corresponding loading factor plots contributing the separation observed in the 2D-PLS-DA, 2D-OPLS-DA, and VIP scores plot resulted in the identification of 9 *m/z* spectral figures with significant discriminatory potential between kidney cancer and controls (Fig. 8d), with color changes depicted on the right-hand side of the VIP scores plot indicating relative higher or lower abundance in the two groups.

Selected *m/z* values were subjected to multivariate ROC curve analyses based on the random forest algorithms. Fifteen *m/z* values were identified with high AUC values with an area under the curve, > 0.75. Eight serum features were putatively identified as known metabolites. Putative identifications were guided by searches on the Human Metabolome Database (HMDB) [30]. All of the identification results are shown in Table 2. The distribution of abundance values of these mass spectral features in control and cancer serum samples is shown in Fig. 4b (selected features) and Fig. S8 (see ESM). Among the metabolites, the most significant was observed for monoglyceride MG(16:0) AUC value (0.82), sensitivity (0.8), and specificity (0.8). As shown in Fig. S9 (see ESM), very good classification was obtained with AUC of 0.963 which is within the range of 0.919 to 1 at the 95% confidence interval (CI). This result suggests that the nine *m/z* values could be used as diagnostic variables enabling the distinction between kidney cancer and the control groups with high specificity and sensitivity.

**Table 2:** Selected mass spectral features with characteristic *m/z* values, as observed in ^109^AgNPET LDI MS spectra of serum from kidney cancer patients and control volunteers

No.	*m/z*^a^	Putative metabolite	Adduct type	Mass error (ppm)	AUC	VIP	p(corr)[1]	*p* value^c^	FC^d^
1	122.943	UN^b^	–	–	0.87	3.05	− 0.666	2.65E−10	1.6
2	304.268	[FA (20:4)] eicosatetraenoyl amine	[C_20_H_33_NO + H]^+^	14.8	0.76	3.05	− 0.608	7.31E−06	43.1
3	353.264	MG(0:0/16:0/0:0)	[C_19_H_38_O_4_ + Na]^+^	− 6.3	0.83	2.69	− 0.569	2.05E−08	2.1
4	390.166	Phe-Thr-Thr	[C_17_H_25_N_3_O_6_ + Na]^+^	6.3	0.90	3.03	− 0.583	8.19E−12	2.1
5	409.153	Thr-Trp-Cys	[C_18_H_24_N_4_O_5_S + H]^+^	− 2.5	0.83	2.69	0.607	1.55E−08	0.5
6	410.157	Glu-Asp-Phe	[C_18_H_23_N_3_O_8_ + H]^+^	2.9	0.79	2.02	0.501	6.11E−07	0.5
7	425.125	Ala-Cys-Pro-Pro	[C_16_H_26_N_4_O_5_S + K]^+^	− 1.3	0.79	2.44	0.554	7.56E−07	0.5
8	439.171	Glu-Arg-Pro	[C_16_H_28_N_6_O_6_ + K]^+^	1.8	0.88	3.08	− 0.631	3.73E−11	2.3
9	467.201	His-Ser-Ser-His	[C_18_H_26_N_8_O_7_ + H]^+^	1.6	0.78	2.47	− 0.568	9.68E−07	4.3

^a^Experimental monoisotopic neutral mass; ^b^Unknown; ^c^*p* value determined from Student’s *t* test with Welch’s correction; ^d^Fold change between kidney cancer and healthy controls calculated from the abundance mean values of each group

## Discussion

### Biological changes associated with potential biomarkers of kidney cancer

Kidney cancer is one of the most studied neoplasms characterized by metabolic reprogramming. Changes in cellular metabolism are reflected in the concentration changes of detected metabolites. The best material for biomarker and metabolomics research is cell cultures and tissues. However, tissue samples must be obtained in invasive way (surgery, biopsy). Therefore, body fluids like urine and serum, which can be obtained with minimal discomfort, are the most preferable materials for biomarker discovery in cancer research. Metabolite levels are in constant flux between tissue and body fluids; therefore, changes in tissue metabolism are reflected in changes in the metabolomes of biofluids. In this study, we analyzed changes in metabolites, which were extracted from serum samples of kidney cancer patients and healthy controls. These data demonstrated that, based of statistically significant (*p* < 0.05) metabolite level changes, cancer patient versus control groups could be clearly differentiated.

Some of the most notable changes were seen in the levels of glucose and lactate, which are substrate and product of glycolysis. In our study, we observed a significant increase in relative glucose concentration in the serum of RCC patients. Intriguingly, these results are inconsistent with the reports of Gao et al.. who showed decreased serum glucose and increased serum lactate in RCC [21]. What is interesting, ^1^H NMR profiling of serum samples from patients with RCC presented by Zira et al. showed that glucose levels were not altered in the RCC patients compared with controls [9]. Increased level of glucose was observed in kidney cancer tissue and urine from patients with RCC in number of previous MS and NMR-based metabolomics studies of kidney cancer [31–33]. Some studies suggest that elevation of serum glucose may be due to a phenomenon called stress-induced hyperglycemia. As Palermo et al.. reported, stress associated with surgery increases sympathetic stimulation and a subsequent rise in catecholamines, cortisol, glucagon, and growth hormone levels [34]. This release of hormones leads to increased serum glucose via gluconeogenesis and glycogenolysis. Moreover, in perioperative period, transient insulin resistance has been observed. Increased level of glucose in malignant cells may be also associated with increasing glycolytic flux caused by an upregulation of numerous glycolysis-related genes in the majority of human cancers [35].

Due to increased aerobic glycolysis, cancer cells tend to produce high amounts of lactate, which is then excreted extracellularly and released into the bloodstream. In our study, we observed decreased level of lactate in serum. It can be explained by the presence of the Cori cycle in the liver which recycles muscle lactate back to glucose, which is then transported back to the muscle for energy production. Moreover, there is emerging evidence that cancer cells may use excess lactate as a fuel for mitochondrial oxidative phosphorylation in a process called lactate shuttling [36]. Lower concentrations of lactate in RCC serum are consistent with the findings of Zira et al. [9], which contrast with Zheng et al.’s report of higher serum lactate levels in RCC patients [11]. Also, metabolic profiling of human RCC tissue and urine showed statistically significantly altered level of lactate in RCC tumors compared with controls [31–33].

In our study, RCC patients exhibited decreased serum levels of glycine and leucine, which may be a reflection of an increased energetic and metabolic demand of proliferating tumor cells for amino acids. The need for increased amino acid pools would be particularly appropriate for essential amino acids (i.e., leucine) that are not synthesized de novo by humans. Our results do not coincide with previous NMR-based serum metabolomics studies that described the increased leucine levels from RCC patients [9, 11]. Regarding the glycine, its decreased level has also been found in urine of patients with RCC which is consistent with our results [37].

Regarding lipid metabolism, choline levels were decreased in the serum samples of RCC patients compared with controls, which may be a consequence of increased choline uptake by cancer cells to fuel phospholipid production for cell membrane formation. Similar results were reported by Gao et al. who observed, in two separate studies, decreased level of serum choline and increased concentration of choline in RCC tissue [12, 21]. Moreover, the reduced level of choline in serum of patients with RCC compared with controls was also reported by Zheng et al. [11]. Previously, Zira show that level of serum choline was increased in RCC patients compared with controls [9].

In this study, we observed decreased level of glycerol in serum samples of RCC patients. Glycerol is a precursor for the synthesis of triacylglycerols and phospholipids, the latter being major structural components of cellular membranes. Moreover, during prolonged fast or in a nutrient-depleted environment (cancer cachexia), glycerol and other substrates (lactate and α-keto acids) may be transformed in liver and kidney into glucose through gluconeogenesis. This finding is also supported by the research of Catchpole et al. who has shown that glycerol is lower in abundance in RCC tissue [38].

*myo*-Inositol, a carbocyclic sugar, is a precursor of secondary messengers including inositol triphosphate and phosphatidylinositol, which mediate cell signal transduction in response to hormones, neurotransmitters, and growth factors. In our study, and when comparing the kidney cancer group to controls, *myo*-inositol levels were found to be decreased in the serum of RCC patients. Similar results concerning the level of *myo*-inositol were reported by Popławski et al. and Catchpole et al. [38]. The research carried out by these teams focused on metabolic differences between RCC and healthy tissue. This convergence of results between ours and theirs supports the notion that changes in cancer tissue metabolism are reflected in distinct changes in metabolite levels in the sera of these patients.

Other metabolites that were found in lower abundance in the sera RCC patients included 1-methylhistidine. Prior studies conducted by Felagan et al. have also confirmed that level of 1-methylhistidine is found to be decreased in the urine of kidney cancer patients [39]. 1-Methylhistidine is considered biomarker of meat consumption [40]. High consumption of red meat and processed meat is associated with increased risk of cancer. However, to date, a direct relationship between 1-methylhistidine and cancerogenesis has not been established.

## Conclusion

This work has demonstrated that high-resolution ^1^H NMR and ^109^AgNPET LDI MS, along with multivariate statistics, are useful approaches to characterize the serum metabolome differences between patients with kidney cancer and healthy people and to separate these two groups based on their distinct serum metabolite profiles. Moreover, ^1^H NMR enabled to identify trends between tumor grades and to discriminate between different types and grades of kidney cancer and healthy controls. With regard to biomarker discovery, 8 potentially robust metabolic biomarkers in 50 serum samples of kidney cancer patients and 49 controls were identified using ^1^H NMR spectroscopy, while 9 mass spectral features were obtained by LDI MS. The most important endogenous compounds having bioactive properties and pharmacological applicability were discussed in details. Both methods have a valuable potential for use as diagnostics and analysis of serum metabolic profiles may be a useful, less invasive way to screen patients with kidney cancer.

## Electronic supplementary material

ESM 1 (PDF 2697 kb).

## Data Availability

The data that support the findings of this study are available from the corresponding author upon reasonable request.

## References

[1] Bray F, Ferlay J, Soerjomataram I, Siegel RL, Torre LA, Jemal A (2018). Global cancer statistics 2018: GLOBOCAN estimates of incidence and mortality worldwide for 36 cancers in 185 countries. CA Cancer J Clin.

[2] Linehan WM, Walther MM, Zbar B (2003). The genetic basis of cancer of the kidney. J Urol.

[3] Hsieh JJ, Purdue MP, Signoretti S, Swanton C, Albiges L, Schmidinger M, Heng DY, Larkin J, Ficarra V (2017). Renal cell carcinoma (RCC) encompasses a heterogeneous group of cancers derived from renal tubular epithelial cells. Nat Publ Group.

[4] Ljungberg B, Hanbury DC, Kuczyk MA, Merseburger AS, Mulders PFA, Patard JJ, Sinescu IC (2007). Renal cell carcinoma guideline. Eur Urol.

[5] Lim W, Graves, Hessamodini, Wong (2013). Metastatic renal cell carcinoma: update on epidemiology, genetics, and therapeutic modalities. Immunotargets Ther.

[6] Farber NJ, Kim CJ, Modi PK, Hon JD, Sadimin ET, Singer EA (2017). Renal cell carcinoma: the search for a reliable biomarker. Transl Cancer Res.

[7] Lin L, Yu Q, Yan X, Hang W, Zheng J, Xing J, Huang B (2010). Direct infusion mass spectrometry or liquid chromatography mass spectrometry for human metabonomics? A serum metabonomic study of kidney cancer. Analyst..

[8] Lin L, Huang Z, Gao Y, Chen Y, Hang W, Xing J, Yan X (2012). LC-MS-based serum metabolic profiling for genitourinary cancer classification and cancer type-specific biomarker discovery. Proteomics..

[9] Zira AN, Theocharis SE, Mitropoulos D, Migdalis V, Mikros E (2010). 1H NMR metabonomic analysis in renal cell carcinoma: a possible diagnostic tool. J Proteome Res.

[10] Jobard E, Blanc E, Négrier S, Escudier B, Gravis G, Chevreau C, Elena-Herrmann B, Trédan O (2015). A serum metabolomic fingerprint of bevacizumab and temsirolimus combination as first-line treatment of metastatic renal cell carcinoma. Br J Cancer.

[11] Zheng H, Ji J, Zhao L, Chen M, Shi A, Pan L, Huang Y, Zhang H, Dong B, Gao H (2016). Prediction and diagnosis of renal cell carcinoma using nuclear magnetic resonance-based serum metabolomics and self-organizing maps. Oncotarget..

[12] Gao H, Dong B, Jia J, Zhu H, Diao C, Yan Z, Huang Y, Li X (2012). Application of ex vivo 1H NMR metabonomics to the characterization and possible detection of renal cell carcinoma metastases. J Cancer Res Clin Oncol.

[13] Misra BB, Upadhayay RP, Cox LA, Olivier M (2018). Optimized GC–MS metabolomics for the analysis of kidney tissue metabolites. Metabolomics..

[14] Jirásko R, Holčapek M, Khalikova M, Vrána D, Študent V, Prouzová Z, Melichar B (2017). MALDI orbitrap mass spectrometry profiling of dysregulated sulfoglycosphingolipids in renal cell carcinoma tissues. J Am Soc Mass Spectrom.

[15] Dill AL, Eberlin LS, Zheng C, Costa AB, Ifa DR, Cheng L, Masterson TA, Koch MO, Vitek O, Cooks RG (2010). Multivariate statistical differentiation of renal cell carcinomas based on lipidomic analysis by ambient ionization imaging mass spectrometry. Anal Bioanal Chem.

[16] Holcakova J, Hernychova L, Bouchal P, Brozkova K, Zaloudik J, Valik D, Nenutil R, Vojtesek B (2008). Identification of αB-crystallin, a biomarker of renal cell carcinoma by SELDI-TOF MS. Int J Biol Markers.

[17] Gil AM, Guedes de Pinho P, Monteiro MS, Duarte IF (2015). NMR metabolomics of renal cancer: an overview. Bioanalysis..

[18] Monteiro MS, Carvalho M, Bastos ML, Guedes de Pinho P (2013). Metabolomics analysis for biomarker discovery: advances and challenges. Curr Med Chem.

[19] Gupta A, Nath K, Bansal N, Kumar M (2020). Role of metabolomics-derived biomarkers to identify renal cell carcinoma: a comprehensive perspective of the past ten years and advancements. Expert Rev Mol Diagn.

[20] Lin L, Huang Z, Gao Y, Yan X, Xing J, Hang W (2011). LC-MS based serum metabonomic analysis for renal cell carcinoma diagnosis, staging, and biomarker discovery. J Proteome Res.

[21] Gao H, Dong B, Liu X, Xuan H, Huang Y, Lin D (2008). Metabonomic profiling of renal cell carcinoma: high-resolution proton nuclear magnetic resonance spectroscopy of human serum with multivariate data analysis. Anal Chim Acta.

[22] Hájek R, Lísa M, Khalikova M, Jirásko R, Cífková E, Študent V, Vrána D, Opálka L, Vávrová K, Matzenauer M, Melichar B, Holčapek M (2018). HILIC/ESI-MS determination of gangliosides and other polar lipid classes in renal cell carcinoma and surrounding normal tissues. Anal Bioanal Chem.

[23] Cífková E, Holčapek M, Lísa M, Vrána D, Melichar B, Študent V (2015). Lipidomic differentiation between human kidney tumors and surrounding normal tissues using HILIC-HPLC/ESI–MS and multivariate data analysis. J Chromatogr B.

[24] Ganti S, Taylor SL, Kim K, Hoppel CL, Guo L, Yang J, Evans C, Weiss RH (2012). Urinary acylcarnitines are altered in human kidney cancer. Int J Cancer.

[25] Kind T, Tolstikov V, Fiehn O, Weiss RH (2007). A comprehensive urinary metabolomic approach for identifying kidney cancer. Anal Biochem.

[26] Nizioł J, Rode W, Laskowska B, Ruman T (2013). Novel Monoisotopic ^109^ AgNPET for laser desorption/ionization mass spectrometry. Anal Chem.

[27] Nizioł J, Ossoliński K, Ossoliński T, Ossolińska A, Bonifay V, Sekuła J, Dobrowolski Z, Sunner J, Beech I, Ruman T (2016). Surface-transfer mass spectrometry imaging of renal tissue on gold nanoparticle enhanced target. Anal Chem.

[28] Emwas AH, Saccenti E, Gao X, McKay RT, dos Santos VAPM, Roy R, Wishart DS (2018). Recommended strategies for spectral processing and post-processing of 1D ^1^H-NMR data of biofluids with a particular focus on urine. Metabolomics..

[29] Chong J, Wishart DS, Xia J (2019). Using MetaboAnalyst 4.0 for comprehensive and integrative metabolomics data analysis. Curr Protoc Bioinformatics.

[30] Wishart DS, Tzur D, Knox C, Eisner R, Guo AC, Young N, Cheng D, Jewell K, Arndt D, Sawhney S, Fung C, Nikolai L, Lewis M, Coutouly M-A, Forsythe I, Tang P, Shrivastava S, Jeroncic K, Stothard P, Amegbey G, Block D, Hau DD, Wagner J, Miniaci J, Clements M, Gebremedhin M, Guo N, Zhang Y, Duggan GE, MacInnis GD, Weljie AM, Dowlatabadi R, Bamforth F, Clive D, Greiner R, Li L, Marrie T, Sykes BD, Vogel HJ, Querengesser L (2007). HMDB: the Human Metabolome Database. Nucleic Acids Res.

[31] Wettersten HI, Hakimi AA, Morin D, Bianchi C, Johnstone ME, Donohoe DR, Trott JF, Aboud OA, Stirdivant S, Neri B, Wolfert R, Stewart B, Perego R, Hsieh JJ, Weiss RH (2015). Grade-dependent metabolic reprogramming in kidney cancer revealed by combined proteomics and metabolomics analysis. Cancer Res.

[32] Popławski P, Tohge T, Bogusławska J, Rybicka B, Tański Z, Treviño V, Fernie AR, Piekiełko-Witkowska A (1863). Integrated transcriptomic and metabolomic analysis shows that disturbances in metabolism of tumor cells contribute to poor survival of RCC patients. Biochim Biophys Acta.

[33] Ragone R, Sallustio F, Piccinonna S, Rutigliano M, Vanessa G, Palazzo S, Lucarelli G, Ditonno P, Battaglia M, Fanizzi F, Schena F (2016). Renal cell carcinoma: a study through NMR-based metabolomics combined with transcriptomics. Diseases..

[34] Palermo NE, Gianchandani RY, McDonnell ME, Alexanian SM (2016). Stress hyperglycemia during surgery and anesthesia: pathogenesis and clinical implications. Curr Diab Rep.

[35] Chesney J, Mojesky A, Imbert-Fernandez Y, Trent J, Telang S. Abstract 5487: targeting glycolysis for the treatment of cancer. In: Cancer Res. 2018;78:5487–5487.

[36] Goodwin ML, Gladden LB, Nijsten MWN, Jones KB (2015). Lactate and cancer: revisiting the Warburg effect in an era of lactate shuttling. Front Nutr.

[37] Monteiro MS, Barros AS, Pinto J, Carvalho M, Pires-Luís AS, Henrique R, Jerónimo C, Bastos MDL, Gil AM, Guedes De Pinho P (2016). Nuclear magnetic resonance metabolomics reveals an excretory metabolic signature of renal cell carcinoma. Sci Rep.

[38] Catchpole G, Platzer A, Weikert C, Kempkensteffen C, Johannsen M, Krause H, Jung K, Miller K, Willmitzer L, Selbig J, Weikert S (2011). Metabolic profiling reveals key metabolic features of renal cell carcinoma. J Cell Mol Med.

[39] Falegan O, Ball M, Shaykhutdinov R, Pieroraio P, Farshidfar F, Vogel H, Allaf M, Hyndman M (2017). Urine and serum metabolomics analyses may distinguish between stages of renal cell carcinoma. Metabolites..

[40] Mitry P, Wawro N, Rohrmann S, Giesbertz P, Daniel H, Linseisen J (2019). Plasma concentrations of anserine, carnosine and pi-methylhistidine as biomarkers of habitual meat consumption. Eur J Clin Nutr.

