# Supplementary material for: Nuclear magnetic resonance and surface-assisted laser desorption/ionization mass spectrometry-based serum metabolomics of kidney cancer
Source: Anal Bioanal Chem. 2020 Jul 13;412(23):5827–41. doi: 10.1007/s00216-020-02807-1 (PMC7413895; doi:10.1007/s00216-020-02807-1)
Supplement: Supplementary file 1 — (PDF 2697 kb). [file 216_2020_2807_MOESM1_ESM.pdf]

Analytical and Bioanalytical Chemistry

Electronic Supplementary Material

Nuclear magnetic resonance and surface-assisted laser desorption/ionization mass spectrometry-based serum metabolomics of kidney cancer

Joanna Nizioł, Krzysztof Ossoliński, Brian P. Tripet, Valérie Copié, Adrian Arendowski, Tomasz Ruman

Table of contents

Table S1 Clinical characteristic of kidney cancer patients **3**

Fig. S1 2D PLS-DA and OPLS-DA between patients with kidney cancer and healthy control based on ^{1}H NMR data set **6**

Table S2 Mean metabolite concentrations for controls *vs*. kidney cancer serum extracts on ^{1}H NMR data set **7**

Fig. S2 ROC curve analysis for potential biomarkers predicted by classical univariate analysis of ^{1}H NMR data..**9**

Fig. S3 Receiver operating curve (ROC) illustrating the performance of the NMR models in distinguishing between patients with kidney cancer and healthy volunteers using 8 metabolite biomarkers **10**

Fig. S4 PLS-DA classification between different types and grades of kidney cancer based on the ^{1}H NMR dataset **11**

Fig. S5 Box-and-whisker plots for different tumor grades based on metabolites detected with ^{1}H NMR..**12**

Fig. S6 Box-and-whisker plots for different tumor types based on metabolites detected with ^{1}H NMR..**12**

Fig. S7 PLS-DA classification between patients with kidney cancer and healthy control on the ^{109}AgNPET LDI MS dataset **14**

Table S3 Mean metabolite abundance for controls vs. kidney cancer serum extracts based on LDI MS data set. **15**

Fig. S8 ROC curve analysis for potential biomarkers predicted by classical univariate analysis with ^{109}AgNPET LDI MS **18**

Fig. S9 Receiver operating curve (ROC) illustrating the performance of the ^{109}AgNPET LDI MS models in distinguishing between patients with kidney cancer and healthy volunteers using 9 variables **19**

Table S1 Clinical characteristic of kidney cancer patients

Patient no.	Histopathological diagnosis of kidney cancer	Size	Sex	Age	Staging	Grading
1	ccRCC	74x66x71	M	61	pT1aN0M0	G4
2	ccRCC	63x42x60	M	89	pT4N0M1 (M:adrenal gland)	G3
3	ccRCC	118x89x105	M	74	pT3aN0M0, invades perirenal fat, invades renal vein	G3
4	ccRCC	51x54x82	M	73	pT3aN0M1 (M:adrenal gland, spinal cord), invades perirenal fat, invades renal vein	G4
5	ccRCC	74x61x72	M	72	pT1bN0M0	G2
6	pRCC	68x60x60	M	78	pT1bN0M0	G2
7	meta – lung adenocarcinoma	70x80x50	M	70	-	-
8	-	31x25x30	M	44	-	-
9	ccRCC	118x82x130	F	57	pT3aN0M1 (M:liver, lung), invades renal vein	G2
10	chRCC	55x72x61	M	73	pT2aN0M0	G3
11	ccRCC	41x30x39	F	87	pT3aN0M0	G3
12	ccRCC	55x51x57	M	77	pT3aN0M0, invades perirenal fat	G3
13	ccRCC	23x26x20	F	69	pT1aN0M0	G2
14	pRCC	17x14x15	M	75	pT1aN0M0	G3
15	AML	23x24x32	M	61	pT1aN0M0	-

16	ccRCC	22x20x20	M	72	pT1aN0M0	G1
17	ccRCC	22x19x25	F	77	pT1aN0M0	G2
18	oncocytoma	25x23x24	F	70	pT1aN0M0	-
19	oncocytoma	27x22x21	M	56	pT1aN0M0	-
20	ccRCC	115x100x135	F	86	pT3aN0M0,<br>invades renal vein	G3
21	ccRCC	20x18x20	M	36	pT1aN0M0	G2
22	ccRCC	25x20x20	M	52	pT1aN0M0	G2
23	ccRCC	26x30x31	F	73	pT1aN0M0	G2
24	CDC	25x25x38	M	70	pT1aN0M1	G3
25	oncocytoma	28x28x30	M	74	pT1aN0M0	-
26	r1:TCRC,<br>r2:AML	R1:25x18x20<br>R2:27x20x20	F	80	R1: pT1aN0M0,<br>R2: pT1aN0M0	-
27	ccRCC	30x20x15	M	57	pT1aN0M0	G1
28	ccRCC	28x24x20	F	81	pT1aN0M0	G2
29	ccRCC	38x33x32	F	65	pT1aN0M0	G2
30	ccRCC	18x17x20	F	66	pT1aN0M0	G1
31	AML	95x58x73	F	53	pT2bN0M0	-
32	ccRCC	20x20x20	M	66	pT1aN0M0	G2
33	ccRCC	46x49x57	F	69	pT3aN1M1,<br>invades renal sinus fat	G3
34	oncocytoma	32x32x31	M	52	pT1aN0M0	-
35	ccRCC	62x76x80	M	80	pT2aN0M0	G3
36	ccRCC	50x34x40	M	70	pT1bN0M0	G2
37	ccRCC	66x48x54	M	73	pT3aN0M0,<br>invades renal vein	G2
38	ccRCC	72x80x83	M	76	pT3aN0M0,<br>invades renal vein	G3
39	ccRCC	20x20x20	M	74	pT1aN0M0	G3
40	SRC	54x50x40	F	78	-	-
41	ccRCC	21x20x20	M	63	pT1aN0M0	G2

42	ccRCC	61x71x60	F	-	pT1bN0M0	G1
43	ccRCC	64x49x50	F	78	pT3aN0M0, invades renal vein	G3
44	ccRCC	23x19x20	M	51	pT1aN0M0	G2
45	ccRCC	35x30x30	F	54	pT1aN0M0	G2
46	ccRCC	40x36x40	F	81	pT1aN0M0	G1
47	chRCC	25x20x20	M	85	pT1aN0M0	G1
48	ccRCC	33x30x30	F	81	pT1aN0M0	G1
49	AML	12x17x12	F	56	pT1aN0M0	-
50	ccRCC	37x32x35	M	68	pT1aN0M0	G2

AML – angiomyolipoma; ccRCC - clear cell RCC; CDC - collecting duct carcinoma chRCC - chromophobe renal cell carcinoma; F – female; N0 - no nodal involvement; N1 - metastatic involvement of regional lymph node(s); M – male; M0 - no distant metastases; M1 - distant metastases; pRCC - papillary RCC; meta – metastasis; SRC - simple renal cyst; T1a - tumor confined to kidney, <4 cm; T1b - tumor confined to kidney, >4 cm but <7 cm; T2a - tumor confined to kidney, >7 cm but not >10 cm; T2b - tumor confined to kidney, >10 cm; T3a: Tumor grossly extends into the renal vein or its segmental branches, or tumor invades perirenal and/or renal sinus fat but not beyond the Gerota fascia; T4: involves ipsilateral adrenal gland or invades beyond Gerota's fascia; TCRC - tubulocystic renal cell carcinoma

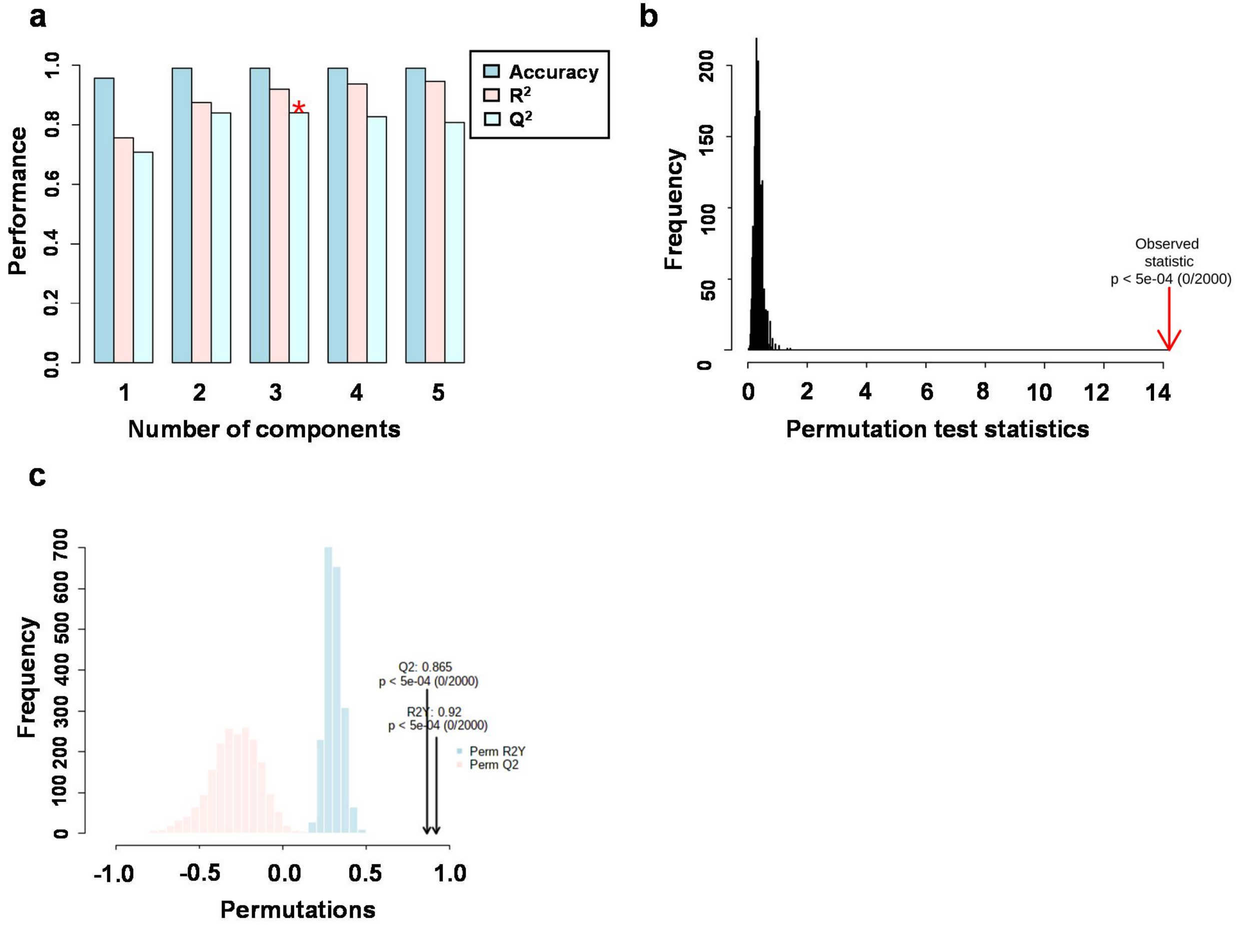

Fig. S1 2D PLS-DA and OPLS-DA based on ^1H NMR data set (a). Supervised PLS-DA classification of kidney cancer patients and healthy controls using different number of components. red star indicates the best classifier, R^2=0.92, Q^2=0.86, accuracy=0.99; (b) Permutation tests based on separation distance of PLS-DA, indicating that the discriminatory power of the PLS-DA model is robust and is associated with a statistically significant p value < 5E-04 (0/2000). (c) A permutation test performed with 2000 random permutations in an OPLS-DA model showing R^2Y= 0.92 and Q^2=0.865

Table S2 Mean metabolite concentrations (mM) for controls vs. kidney cancer serum extracts on ^{1}H NMR data set. Those metabolites highlighted in bold are considered statistically significantly different ($p < 0.05$; FDR < 0.05; VIP > 1; |p(corr)| > 0.5) between controls and kidney cancer serum extracts

No.	Name	HMDB ID	Control		Cancer		p-value	q-value (FDR)	Fold Change	VIP	AUC	p[1]	p(corr)[1]
			Mean	SD	Mean	SD							
1	2-Oxoisocaproate	HMDB0000695	0.0054	0.0029	0.0074	0.0035	4.54E-02	8.14E-02	1.25	0.59	0.62	-0.43	-0.194
2	3-Hydroxybutyrate	HMDB0000357	0.0241	0.0352	0.0632	0.0814	5.89E-03	1.27E-02	2.47	0.96	0.66	-0.74	-0.336
3	3-Methyl-2-oxovalerate	HMDB0000491	0.0049	0.0020	0.0062	0.0024	1.06E-01	1.63E-01	1.16	0.53	0.60	-0.34	-0.154
4	Acetate	HMDB0000042	0.3633	0.0270	0.3535	0.0323	3.96E-02	7.41E-02	0.90	0.61	0.62	0.40	0.181
5	Acetone	HMDB0001659	0.2042	0.1313	0.1877	0.0558	6.01E-01	6.47E-01	0.82	0.06	0.53	-0.04	-0.018
6	Alanine	HMDB0000161	0.1170	0.0302	0.1010	0.0310	2.86E-04	7.23E-04	0.81	1.14	0.71	0.94	0.426
7	Asparagine	HMDB0000168	0.0161	0.0038	0.0144	0.0033	1.15E-02	2.36E-02	0.82	0.75	0.65	0.50	0.224
8	Aspartate	HMDB0000191	0.0060	0.0030	0.0043	0.0012	4.91E-04	1.17E-03	0.68	0.53	0.70	0.37	0.170
9	Betaine	HMDB0000043	0.0184	0.0077	0.0160	0.0049	3.70E-03	8.36E-03	0.88	0.25	0.67	-0.15	-0.067
10	Carnitine	HMDB0000062	0.0146	0.0090	0.0169	0.0045	9.86E-01	9.86E-01	1.11	0.91	0.50	-0.66	-0.297
**11**	**Choline**	**HMDB0000097**	**0.0063**	**0.0032**	**0.0045**	**0.0010**	**1.04E-08**	**8.92E-08**	**0.70**	**1.76**	**0.84**	**1.35**	**0.609**
12	Creatine	HMDB0000064	0.0117	0.0055	0.0116	0.0090	5.37E-02	9.23E-02	0.88	0.46	0.61	0.43	0.192
13	Creatinine	HMDB0000562	0.0409	0.0516	0.0381	0.0162	2.51E-01	3.08E-01	0.97	0.16	0.57	-0.15	-0.070
14	Dimethyl sulfone	HMDB0004983	0.0029	0.0017	0.0030	0.0013	8.79E-01	9.21E-01	0.97	0.19	0.51	-0.15	-0.069
15	dss	-	0.2432	0.0088	0.2454	0.0009	2.31E-01	2.92E-01	0.94	0.36	0.57	0.21	0.097
16	Formate	HMDB0000142	0.0055	0.0063	0.0062	0.0087	4.32E-01	5.02E-01	1.04	0.34	0.55	0.27	0.122
**17**	**Glucose**	**HMDB0000122**	**1.1266**	**0.3711**	**1.9326**	**0.7747**	**1.85E-13**	**3.98E-12**	**1.58**	**2.12**	**0.93**	**-1.57**	**-0.708**
18	Glutamine	HMDB0000641	0.1011	0.0308	0.0997	0.0304	2.28E-01	2.92E-01	0.95	0.41	0.57	0.38	0.173
**19**	**Glycerol**	**HMDB0000131**	**0.0356**	**0.0122**	**0.0266**	**0.0247**	**1.48E-07**	**7.06E-07**	**0.70**	**1.49**	**0.81**	**1.14**	**0.516**
**20**	**Glycine**	**HMDB0000123**	**0.0800**	**0.0231**	**0.0605**	**0.0164**	**1.93E-07**	**8.31E-07**	**0.71**	**1.58**	**0.81**	**1.17**	**0.531**
21	Glycolate	HMDB0000115	0.0138	0.0081	0.0110	0.0024	3.07E-05	8.81E-05	0.75	0.46	0.74	0.37	0.168
22	Histidine	HMDB0000177	0.0118	0.0061	0.0121	0.0042	4.49E-01	5.08E-01	0.91	0.50	0.54	-0.39	-0.176
23	Hypoxanthine	HMDB0000157	0.0031	0.0018	0.0014	0.0005	4.64E-09	4.99E-08	0.40	1.48	0.84	1.07	0.486
24	Imidazole	HMDB0001525	0.3128	0.0309	0.3577	0.0482	1.95E-01	2.68E-01	1.06	0.36	0.58	-0.27	-0.123
25	Isoleucine	HMDB0000172	0.0169	0.0048	0.0167	0.0035	1.01E-01	1.63E-01	0.92	0.01	0.60	-0.05	-0.023
26	Isovalerate	HMDB0000718	0.0035	0.0032	0.0048	0.0012	1.20E-01	1.79E-01	1.27	1.30	0.59	-0.98	-0.442

**27**	**Lactate**	**HMDB0000190**	**0.8862**	**0.2093**	**0.6060**	**0.3001**	**2.10E-08**	**1.29E-07**	**0.63**	**1.78**	**0.83**	**1.42**	**0.641**
**28**	**Leucine**	**HMDB0000687**	**0.0393**	**0.0066**	**0.0327**	**0.0089**	**8.23E-08**	**4.42E-07**	**0.77**	**1.56**	**0.81**	**1.15**	**0.522**
29	Lysine	HMDB0000182	0.0261	0.0079	0.0187	0.0036	1.93E-08	1.29E-07	0.67	0.71	0.83	0.50	0.228
30	Methionine	HMDB0000696	0.0072	0.0034	0.0078	0.0027	2.00E-01	2.68E-01	0.97	0.57	0.58	-0.34	-0.155
**31**	**myo-Inositol**	**HMDB0000211**	**0.0275**	**0.0095**	**0.0100**	**0.0016**	**3.11E-16**	**1.34E-14**	**0.35**	**2.02**	**0.98**	**1.43**	**0.645**
32	Phenylalanine	HMDB0000159	0.0267	0.0063	0.0196	0.0058	5.14E-10	7.37E-09	0.68	0.73	0.86	0.62	0.279
**33**	**1-Methylhistidine**	**HMDB0000001**	**0.0073**	**0.0062**	**0.0021**	**0.0038**	**2.72E-07**	**1.06E-06**	**0.27**	**1.71**	**0.80**	**1.29**	**0.582**
34	Proline	HMDB0000162	0.0539	0.0265	0.0696	0.0296	1.03E-01	1.63E-01	1.25	0.72	0.60	-0.50	-0.226
35	Propionate	HMDB0000237	0.0081	0.0025	0.0118	0.0025	5.51E-05	1.48E-04	1.36	1.09	0.74	-0.79	-0.359
36	Pyroglutamate	HMDB0000267	0.0374	0.0219	0.0202	0.0178	1.91E-05	5.88E-05	0.53	1.33	0.75	0.95	0.429
37	Pyruvate	HMDB0000243	0.0047	0.0031	0.0054	0.0035	5.29E-01	5.84E-01	1.00	0.27	0.54	-0.21	-0.093
38	Succinate	HMDB0000254	0.0061	0.0049	0.0013	0.0005	7.99E-07	2.86E-06	0.21	1.43	0.79	0.98	0.443
39	Threonine	HMDB0000167	0.0412	0.0139	0.0467	0.0101	1.54E-01	2.21E-01	1.01	0.41	0.58	-0.35	-0.160
40	Tryptophan	HMDB0000929	0.0126	0.0034	0.0137	0.0037	9.74E-01	9.86E-01	1.01	0.24	0.50	-0.17	-0.078
41	Tyrosine	HMDB0000158	0.0220	0.0067	0.0187	0.0060	1.74E-05	5.75E-05	0.81	0.42	0.75	0.35	0.159
42	Urea	HMDB0000294	3.4645	1.8725	3.3771	1.3919	3.11E-01	3.72E-01	0.96	0.27	0.56	0.24	0.110
43	Valine	HMDB0000883	0.0510	0.0107	0.0484	0.0115	1.52E-02	2.98E-02	0.89	0.74	0.64	0.66	0.299

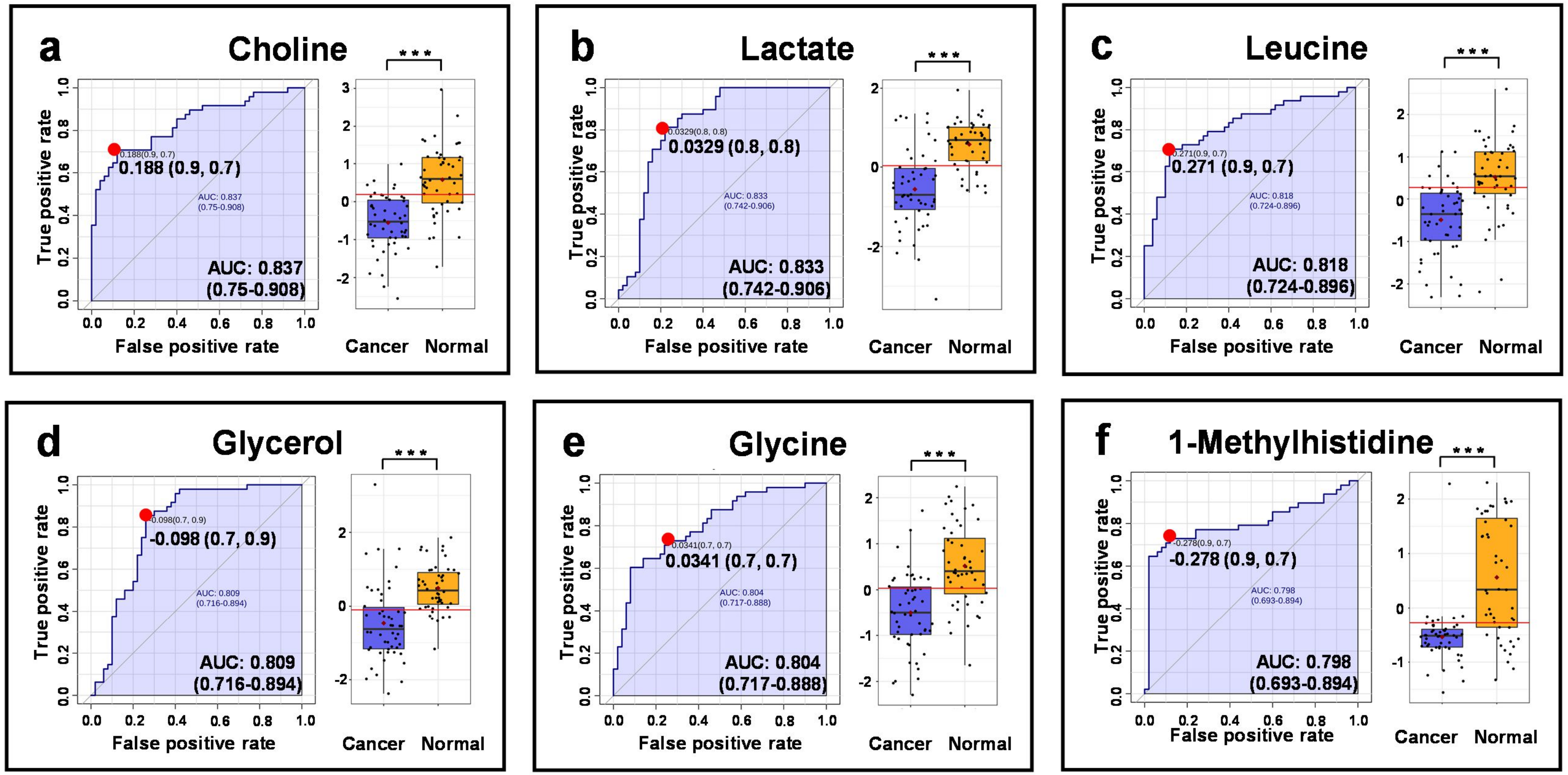

Fig. S2 ROC curve analysis for potential biomarkers predicted by classical univariate analysis by ^{1}H NMR. The left-hand side of each panel indicates ROC curve for a particular metabolite, with 95% confidence interval (shadowed) and the solid red dot indicating the optimal cut-off, associated with sensitivity and specificity values. The right-hand side of each panel depicts the distribution of metabolite level values observed in control and kidney cancer serum samples. The horizontal red line in the graphs indicates the cut-off point

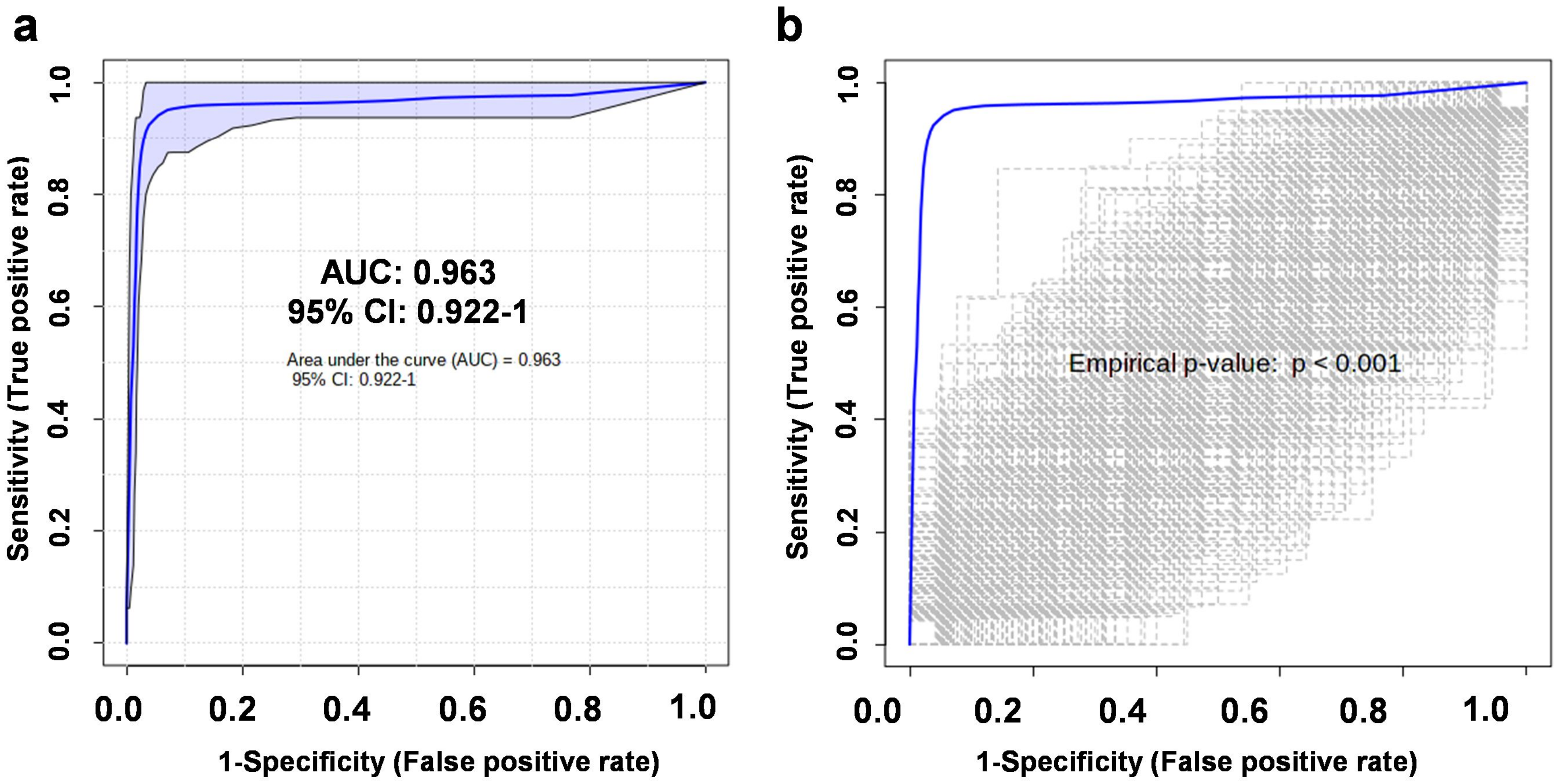

Fig. S3 (a) Receiver operating curve (ROC) illustrating the performance of the NMR models in distinguishing between patients with kidney cancer and healthy volunteers using 8 metabolite biomarker (b) Permutation test based on measure area under ROC curve. The p value based on permutation is $p < 0.001$(0/1000)

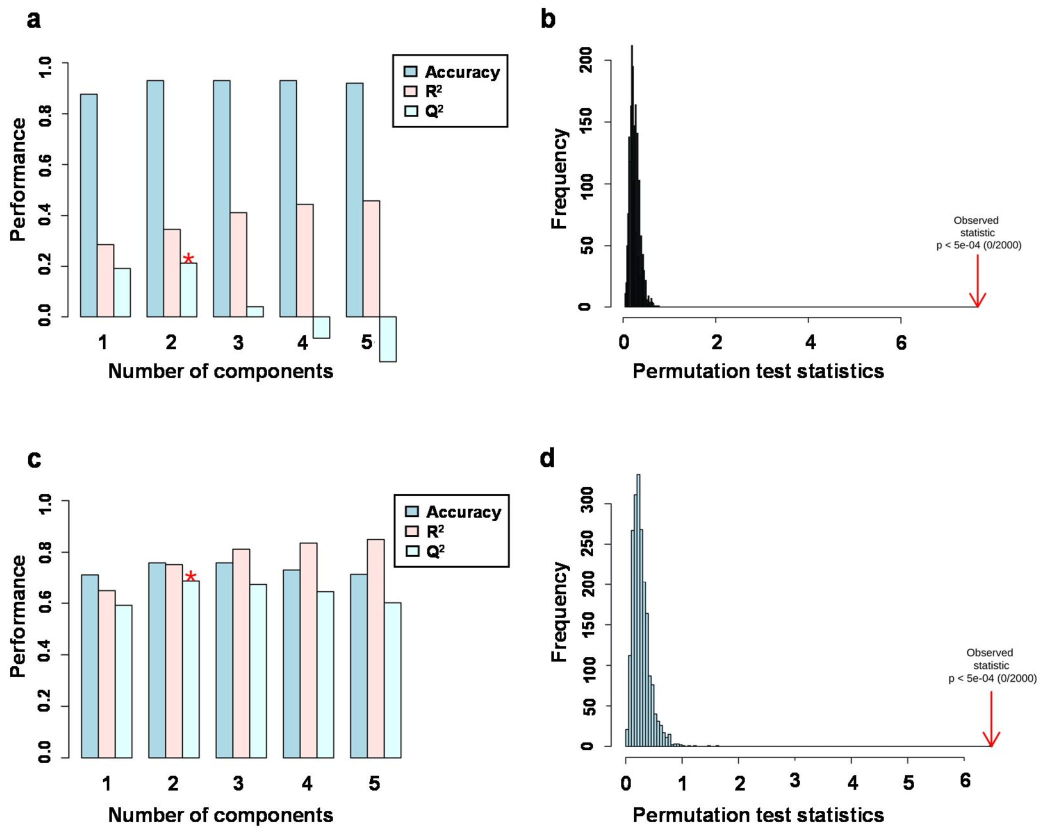

Fig. S4 PLS-DA based on the ^{1}H NMR dataset (a) Supervised 2D PLS-DA classification between malignant, benign kidney cancer and healthy volunteers using different number of components. The red star indicates the best classifier, R^2=0.34, Q^2=0.23, accuracy=0.93; (b) Permutation tests based on separation distance of PLS-DA model for malignant, benign kidney cancer and healthy volunteers. The p value based on permutation is p < 5E-04 (0/2000). (c) Supervised 2D PLS-DA classification between different grades of cancer and healthy volunteers using different number of components. The red circle indicates the best classier, R^2=0.75, Q^2=0.69, accuracy=0.77; (d) Permutation tests based on separation distance of PLS-DA model for different grades of cancer and healthy volunteers, with an associated p value of < 5E-4 (0/2000)

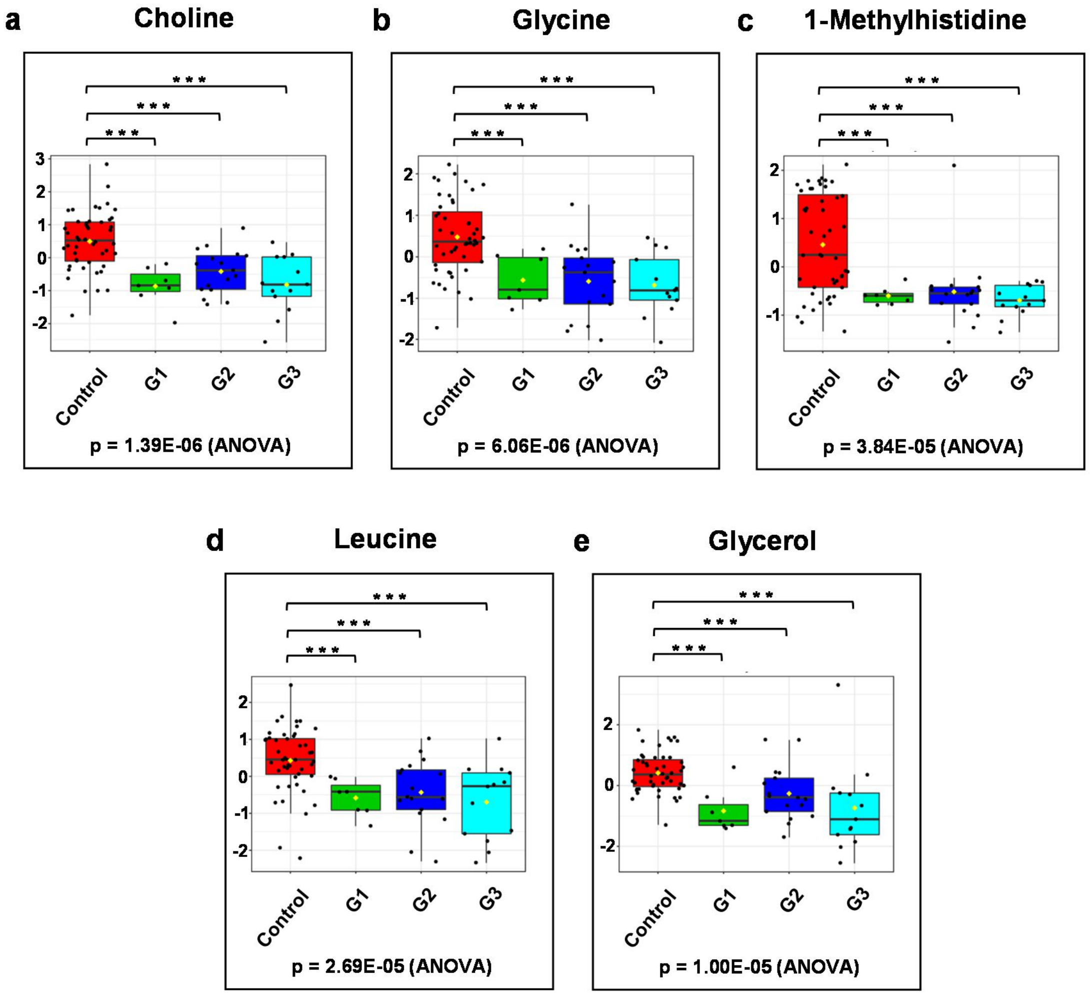

Fig. S5 Box-and-whisker plots for five panels of metabolites detected by ^{1}H NMR whose normalized concentration changes between the different groups are significant (i.e. p-values < 0.05), and illustrating that serum profiles from control and cancer patients change slightly as a function of kidney tumor grade (G1-3), but major changes are primarily between control and kidney cancer groups, irrespective of tumor grades. The horizontal line in the middle portion of each box plot in the figure indicates median concentration values of the select metabolites

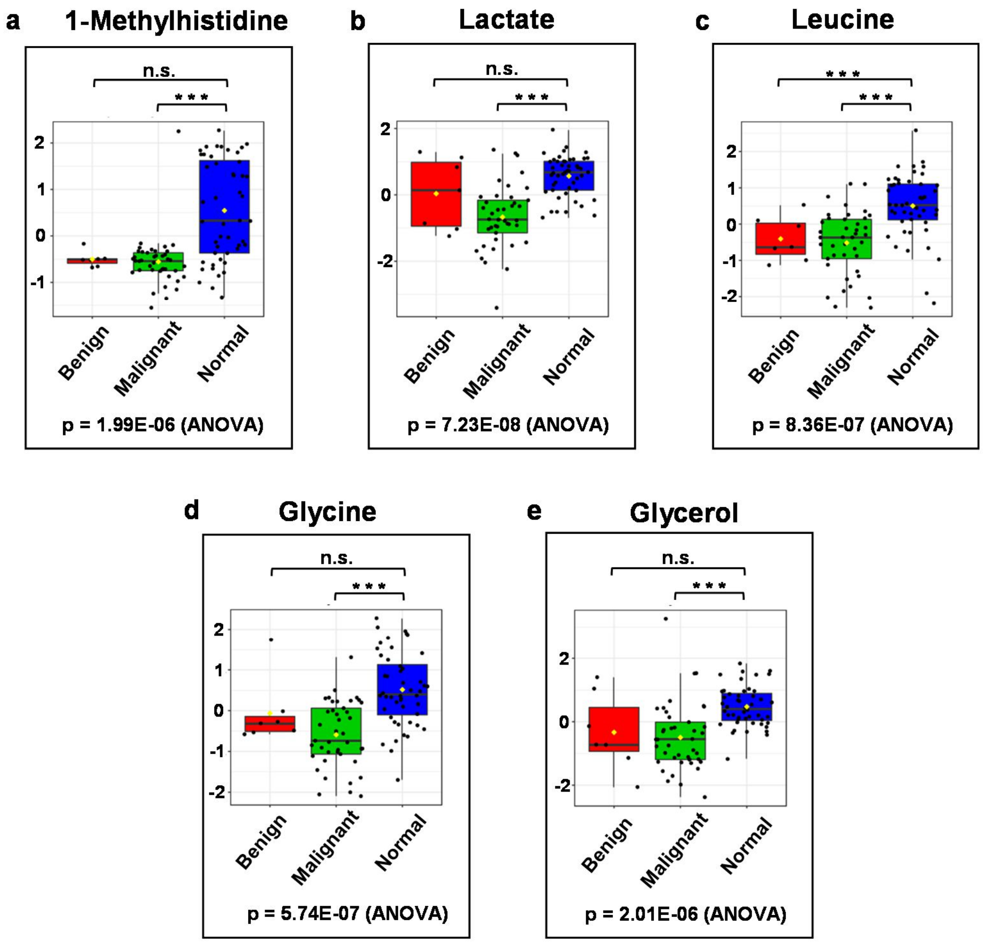

Fig. S6 Box plots of eleven metabolites detected by ^{1}H NMR whose changes in normalized concentrations between benign, malignant, and control groups are significant (i.e. p-values < 0.05). These data indicate that the metabolite profiles of serum extracts from kidney cancer patients and healthy controls separate the different groups based on whether the kidney cancer tumor is benign or malignant. As in Fig 7, the horizontal line in the middle portion of each box represents median normalized metabolite concentration value

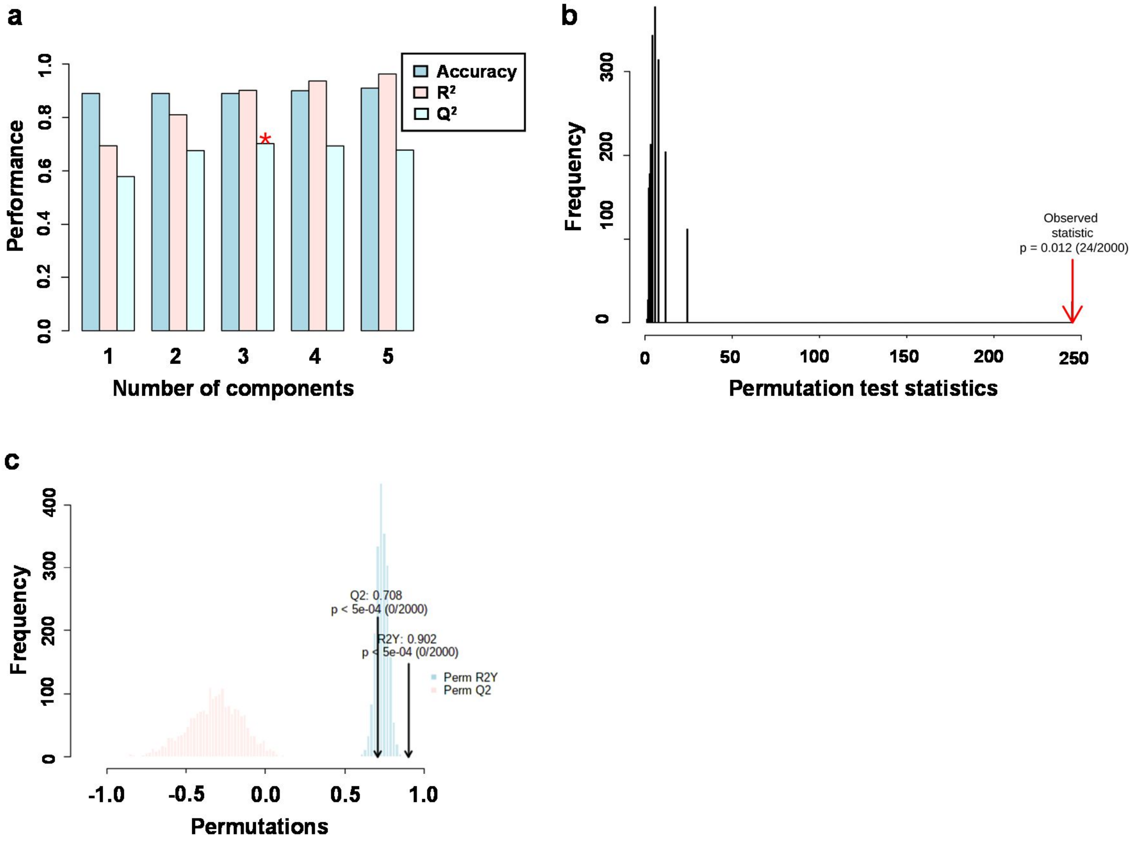

Fig. S7 PLS-DA and OPLS-DA on the [109]AgNPET LDI MS dataset. (a) PLS DA classification using different number of components based. The red circle indicates the best classier, R^2=0.90, Q^2=0.70, accuracy=0.89; (b) Permutation tests based on separation distance, with an associated p value of < 0.012 (24/2000). (c) A permutation test performed with 2000 random permutations in an OPLS-DA model showing R^2Y= 0.902 and Q2=0.708

Table S3 Mean metabolite abundance for controls vs. kidney cancer serum extracts based on LDI MS data set. Those variables highlighted in bold are considered statistically significantly different (p<0.05; FDR < 0.05; VIP>1; AUC>0.75; |p(corr)| > 0.5) between controls and kidney cancer serum extracts

No.	*m/z*	Control		Cancer		p-value	q-value (FDR)	Fold Change	VIP	AUC	p[1]	p(corr)[1]
		Mean	SD	Mean	SD							
1	80.970	20593	5229	18391	5420	1.12E-04	1.09E-03	8.93E-01	1.74	0.72	1.29	0.351
2	90.997	2889	960	4361	1853	6.13E-08	2.39E-06	1.51E+00	2.34	0.81	-1.84	-0.498
3	100.128	686	454	948	438	1.65E-03	9.90E-03	1.38E+00	1.45	0.68	-0.85	-0.230
4	104.125	7107	3021	5376	2038	2.99E-05	4.58E-04	7.56E-01	2.12	0.74	1.70	0.462
5	104.976	11600	3149	12909	3392	1.99E-03	1.15E-02	1.11E+00	1.62	0.68	-1.43	-0.387
**6**	**106.969**	**5391**	**1580**	**8163**	**3281**	**3.31E-10**	**2.58E-08**	**1.51E+00**	**3.09**	**0.86**	**-2.51**	**-0.680**
7	113.041	2195	960	1492	887	2.45E-06	4.79E-05	6.80E-01	1.09	0.77	0.94	0.256
8	120.957	11139	3380	13194	4112	1.25E-04	1.15E-03	1.18E+00	2.02	0.72	-1.67	-0.453
**9**	**122.943**	**5398**	**1771**	**8408**	**3658**	**2.65E-10**	**2.58E-08**	**1.56E+00**	**3.05**	**0.87**	**-2.46**	**-0.666**
10	129.014	1033	272	887	242	7.77E-04	5.27E-03	8.59E-01	1.15	0.70	1.03	0.280
11	134.036	1723	875	1220	608	7.77E-04	5.27E-03	7.08E-01	1.25	0.70	0.88	0.239
12	135.020	17850	6916	13175	6143	3.92E-05	5.10E-04	7.38E-01	2.06	0.74	1.46	0.395
13	136.066	5864	11231	5602	2940	6.28E-05	7.26E-04	9.55E-01	1.26	0.73	-0.85	-0.230
14	147.895	1144	586	809	578	1.12E-04	1.09E-03	7.07E-01	1.12	0.72	0.66	0.178
15	150.991	20670	8048	16228	6871	3.42E-04	2.60E-03	7.85E-01	1.76	0.71	1.20	0.324
16	152.036	5766	12696	6356	4270	7.97E-04	5.29E-03	1.10E+00	1.57	0.69	-1.20	-0.325
17	152.953	3313	1102	2164	1323	7.76E-09	4.84E-07	6.53E-01	1.77	0.84	1.28	0.347
18	163.967	1005	642	503	644	3.08E-05	4.58E-04	5.01E-01	1.85	0.74	1.36	0.370
19	166.961	11225	4823	9455	4360	3.75E-03	2.01E-02	8.42E-01	0.31	0.67	0.20	0.055
20	167.980	1170	405	973	382	3.67E-06	6.74E-05	8.32E-01	0.81	0.77	0.70	0.189
21	176.909	797	537	1106	616	9.41E-05	1.01E-03	1.39E+00	1.08	0.73	-0.80	-0.218
22	177.872	1269	575	1590	489	2.56E-03	1.43E-02	1.25E+00	1.33	0.68	-0.91	-0.248
23	179.998	1199	336	1036	342	1.05E-03	6.66E-03	8.64E-01	0.98	0.69	0.92	0.250
24	185.974	476	457	189	284	2.48E-04	1.99E-03	3.98E-01	1.66	0.71	1.23	0.334
25	191.046	1365	620	922	603	3.33E-04	2.60E-03	6.75E-01	1.55	0.71	1.33	0.360
26	194.129	249	388	569	434	5.32E-04	3.86E-03	2.29E+00	1.95	0.70	-1.45	-0.394

27	197.965	1034	399	764	356	1.04E-06	2.49E-05	7.39E-01	1.59	0.78	1.45	0.393
28	198.943	2417	851	1819	765	1.36E-07	4.72E-06	7.52E-01	1.44	0.81	1.08	0.292
29	201.043	115	249	401	378	1.47E-03	9.14E-03	3.49E+00	1.95	0.68	-1.34	-0.364
30	201.947	379	365	94	219	5.59E-05	6.71E-04	2.48E-01	2.22	0.73	1.70	0.460
31	203.066	63646	21763	81921	33732	1.79E-04	1.55E-03	1.29E+00	1.83	0.72	-1.37	-0.372
32	204.072	2749	1078	3646	1653	1.65E-04	1.47E-03	1.33E+00	1.70	0.72	-1.21	-0.329
33	205.070	1276	2934	1221	670	8.38E-04	5.44E-03	9.56E-01	1.12	0.69	-0.61	-0.165
34	207.020	1430	743	973	615	2.17E-04	1.78E-03	6.80E-01	1.26	0.71	0.95	0.258
35	210.865	2354	865	1995	703	7.71E-03	3.94E-02	8.48E-01	1.14	0.65	0.86	0.233
36	219.041	13594	5783	20283	11242	1.47E-06	3.28E-05	1.49E+00	1.83	0.78	-1.31	-0.356
37	220.043	409	701	1042	1051	2.50E-03	1.42E-02	2.55E+00	1.71	0.68	-1.22	-0.331
38	220.938	4019	1514	2994	1253	2.14E-06	4.46E-05	7.45E-01	1.37	0.78	1.05	0.285
39	221.978	2945	3222	3210	1828	1.84E-04	1.55E-03	1.09E+00	1.19	0.72	-0.71	-0.192
40	226.007	552	364	377	352	6.01E-03	3.18E-02	6.82E-01	1.18	0.66	0.87	0.236
41	232.913	867	341	660	391	3.35E-03	1.84E-02	7.61E-01	1.34	0.67	1.04	0.283
42	240.826	1335	553	1080	566	1.54E-03	9.40E-03	8.09E-01	1.09	0.68	0.87	0.236
43	270.969	765	373	980	441	1.94E-03	1.14E-02	1.28E+00	1.30	0.68	-0.93	-0.251
44	280.037	261	420	676	535	1.30E-05	2.13E-04	2.59E+00	2.23	0.75	-1.60	-0.434
45	304.268	10	72	436	416	7.31E-06	1.27E-04	4.31E+01	3.05	0.76	-2.24	-0.608
46	351.136	552	281	376	284	1.08E-04	1.09E-03	6.81E-01	1.39	0.72	1.12	0.304
**47**	**353.264**	**782**	**409**	**1613**	**1176**	**2.05E-08**	**9.12E-07**	**2.06E+00**	**2.69**	**0.83**	**-2.10**	**-0.569**
48	381.291	88	270	459	1278	5.74E-04	4.07E-03	5.19E+00	1.89	0.70	-1.71	-0.463
49	388.155	138	233	313	370	8.71E-03	4.38E-02	2.27E+00	1.43	0.65	-1.14	-0.308
**50**	**390.166**	**1597**	**724**	**3355**	**1960**	**8.19E-12**	**2.55E-09**	**2.10E+00**	**3.03**	**0.90**	**-2.15**	**-0.583**
**51**	**409.153**	**6650**	**3428**	**3076**	**1550**	**1.55E-08**	**8.04E-07**	**4.63E-01**	**2.69**	**0.83**	**2.24**	**0.607**
**52**	410.157	**971**	**526**	**514**	**301**	**6.11E-07**	**1.91E-05**	**5.29E-01**	**2.02**	**0.79**	**1.85**	**0.501**
53	414.201	508	479	1105	1283	8.15E-05	9.09E-04	2.17E+00	2.16	0.73	-1.54	-0.417
**54**	**425.125**	**2992**	**1612**	**1557**	**815**	**7.56E-07**	**2.15E-05**	5.20E-01	**2.44**	**0.79**	**2.04**	**0.554**
55	426.130	442	325	221	250	3.92E-05	5.10E-04	4.99E-01	1.70	0.74	1.46	0.395
**56**	**439.171**	**884**	**513**	**2077**	**1370**	**3.73E-11**	**5.81E-09**	**2.35E+00**	**3.08**	**0.88**	**-2.32**	**-0.631**
**57**	**467.201**	**145**	**322**	**617**	**1082**	**9.68E-07**	**2.49E-05**	**4.26E+00**	**2.47**	**0.78**	**-2.09**	**-0.568**
58	506.767	270	276	637	620	4.42E-05	5.51E-04	2.36E+00	1.91	0.74	-1.40	-0.380

59	527.184	264	256	76	167	3.92E-05	5.10E-04	2.90E-01	2.13	0.74	1.83	0.496
60	659.269	894	590	1753	1658	6.14E-03	3.19E-02	1.96E+00	1.59	0.66	-1.08	-0.292
61	660.275	209	290	534	502	4.80E-04	3.57E-03	2.55E+00	1.78	0.70	-1.50	-0.406
62	675.248	78	153	308	333	1.21E-04	1.15E-03	3.94E+00	2.07	0.72	-1.63	-0.443

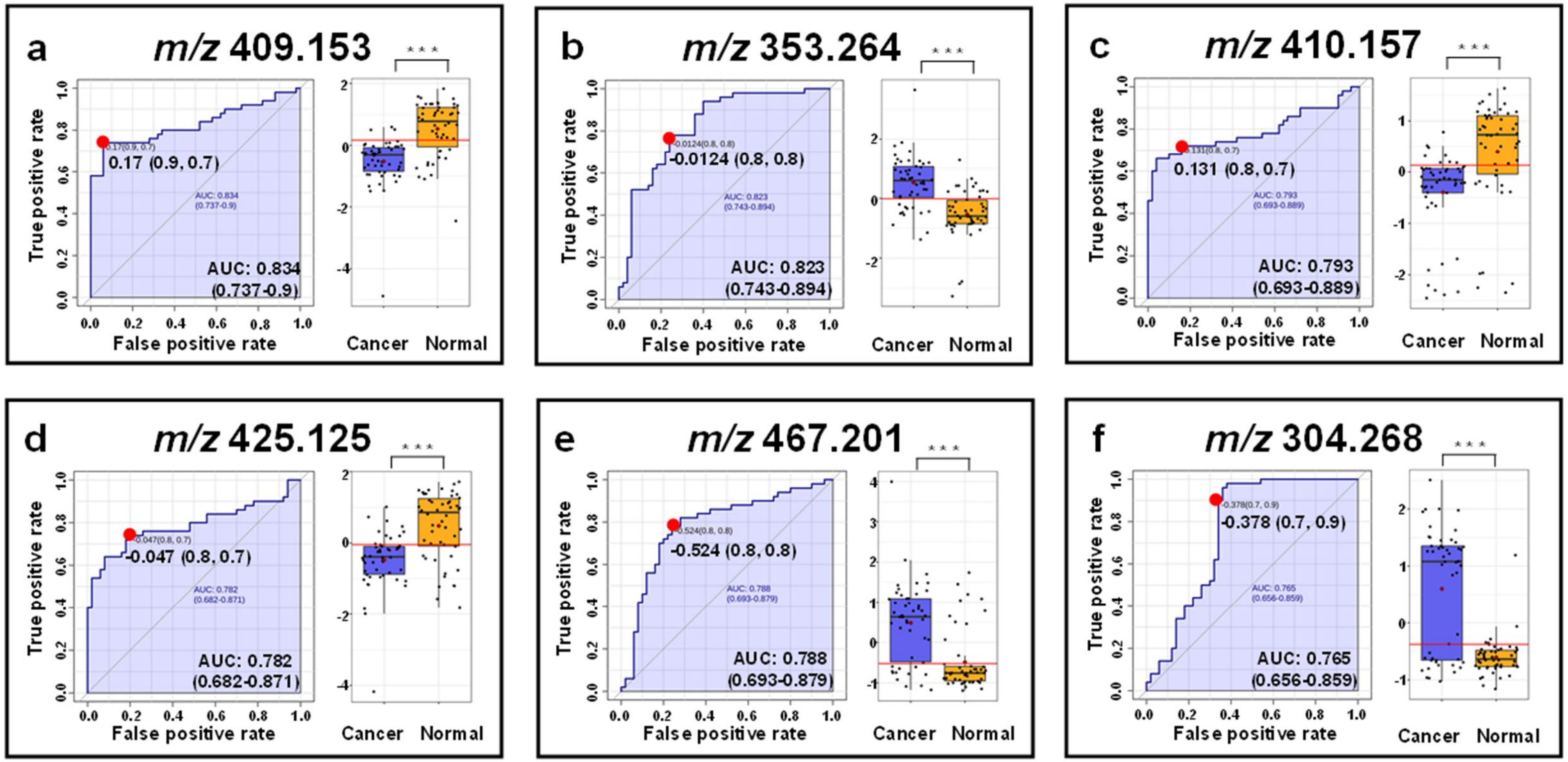

Fig. S8 ROC curve analysis for potential biomarkers predicted by classical univariate analysis by ^{109}AgNPET LDI. The left-hand side of each panel indicates ROC curve for a particular metabolite, with 95% confidence interval (shadowed) and the solid red dot indicating the optimal cut-off, associated with sensitivity and specificity values. The right-hand side of each panel depicts the distribution of metabolite level values observed in control and kidney cancer serum samples. The horizontal red line in the graphs indicates the cut-off point

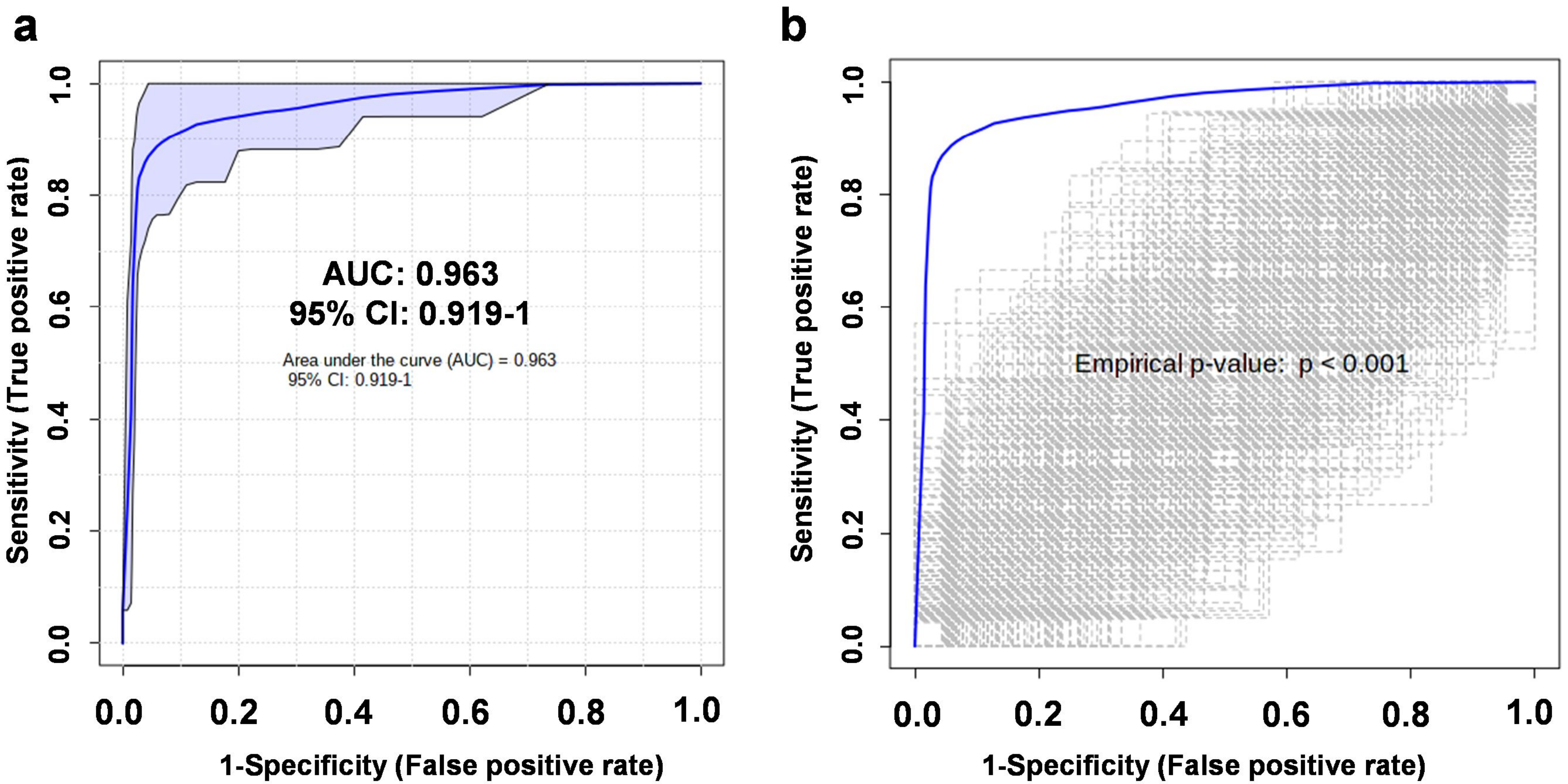

Fig. S9 (a) Receiver operating curve (ROC) illustrating the performance of the LDI MS model in distinguishing between patients with kidney cancer and healthy volunteers using 9 variables (b) Permutation test based on measure area under ROC curve. The p value based on permutation is $p < 0.001(0/1000)$
